# Structural insights into promoter-proximal pausing of RNA polymerase II at +1 nucleosome

**DOI:** 10.1126/sciadv.adu0577

**Published:** 2025-03-05

**Authors:** Masahiro Naganuma, Tomoya Kujirai, Haruhiko Ehara, Tamami Uejima, Tomoko Ito, Mie Goto, Mari Aoki, Masami Henmi, Sayako Miyamoto-Kohno, Mikako Shirouzu, Hitoshi Kurumizaka, Shun-ichi Sekine

**Affiliations:** ^1^ RIKEN Center for Biosystems Dynamics Research, 1-7-22 Suehiro-cho, Tsurumi-ku, Yokohama 230-0045, Japan.; ^2^ Laboratory of Chromatin Structure and Function, Institute for Quantitative Biosciences, The University of Tokyo, 1-1-1 Yayoi, Bunkyo-ku, Tokyo 113-0032, Japan.; ^3^ Department of Biological Sciences, Graduate School of Science, The University of Tokyo, 1-1-1 Yayoi, Bunkyo-ku, Tokyo 113-0032, Japan.

## Abstract

The metazoan transcription elongation complex (EC) of RNA polymerase II (RNAPII) generally stalls between the transcription start site and the first (+1) nucleosome. This promoter-proximal pausing involves negative elongation factor (NELF), 5,6-dichloro-1-β-d-ribobenzimidazole sensitivity-inducing factor (DSIF), and transcription elongation factor IIS (TFIIS) and is critical for subsequent productive transcription elongation. However, the detailed pausing mechanism and the involvement of the +1 nucleosome remain enigmatic. Here, we report cryo–electron microscopy structures of ECs stalled on nucleosomal DNA. In the absence of TFIIS, the EC is backtracked/arrested due to conflicts between NELF and the nucleosome. We identified two alternative binding modes of NELF, one of which reveals a critical contact with the downstream DNA through the conserved NELF-E basic helix. Upon binding with TFIIS, the EC progressed to the nucleosome to establish a paused EC with a partially unwrapped nucleosome. This paused EC strongly restricts EC progression further downstream. These structures illuminate the mechanism of RNAPII pausing/stalling at the +1 nucleosome.

## Teaser

Structural and biochemical analyses elucidate how NELF and the +1 nucleosome are involved in promoter-proximal pausing of RNAPII.

## INTRODUCTION

The eukaryotic genome is housed within the cell nucleus as chromatin, which comprises multiple repetitive units, nucleosomes. Gene expression occurs in this chromatin environment, where DNA-dependent RNA polymerase II (RNAPII) transcribes protein-coding genes and many noncoding genes. Transcription generally consists of three different stages: transcription initiation, elongation, and termination. In metazoans, a regulatory checkpoint exists in the early elongation stage between the transcription start site (TSS) and the first nucleosome immediately downstream of the TSS (the +1 nucleosome). After transcription initiation, RNAPII stalls 20 to 60 base pairs (bp) downstream of the TSS. This phenomenon, called promoter-proximal pausing (or stalling), has been observed in stress-inducible genes and protooncogenes (*1*–*5*). The pausing is now recognized as a universal feature of most metazoan genes and is required to provide a window of opportunity for the early elongation complex (EC) of RNAPII to transition into the productive EC, ensuring accurate and efficient transcription through chromatin.

Two RNAPII-associating factors, negative elongation factor (NELF) and 5,6-dichloro-1-β-d-ribobenzimidazole sensitivity-inducing factor (DSIF), play pivotal roles in the establishment of RNAPII pausing (*6*–*8*). NELF is a large, metazoan-specific protein complex consisting of four subunits, NELF-A, NELF-B, NELF-C/D, and NELF-E. It binds both RNAPII and the nascent RNA transcript emerging from RNAPII and inhibits transcription (*9*). DSIF, a conserved heterodimer complex of Suppressor of Ty 4 (SPT4) and SPT5, is required for NELF recruitment and RNAPII pausing and also serves as a processivity factor upon the pause release by NELF dissociation (*6*, *10*). A previous cryo–electron microscopy (cryo-EM) structure of a paused EC (PEC) of mammalian RNAPII complexed with NELF and DSIF revealed the RNAPII-binding manner and the inhibition mechanism by NELF (*11*): RNAPII is catalytically inactive because its active site holds a “tilted” DNA/RNA hybrid incompatible with RNA elongation, and NELF blocks the binding site of the transcription elongation factor TFIIS, which supports RNA elongation by rescuing stalled ECs due to backtracking (*12*, *13*).

Besides these pausing factors, several lines of evidence have suggested that the chromatin structure plays a critical role in the RNAPII pausing. Chromatin immunoprecipitation (ChIP) experiments in human cells demonstrated that a strongly positioned +1 nucleosome increases RNAPII pausing (*14*). Genome-wide studies revealed that RNAPII stalls near the entry of the +1 nucleosome (*15*–*18*), which seems to participate in the pause maintenance (*19*). A direct contact between RNAPII and the +1 nucleosome was detected in *Drosophila* and human cells (*15*, *17*). Therefore, the mechanisms of RNAPII pausing/stalling and the effects of the chromatin structure remain unclear. To gain structural insights into RNAPII pausing/stalling in the chromatin context, in the present study, we conducted cryo-EM analyses of a mammalian EC stalled on a nucleosomal DNA template. The structures and accompanying biochemical analyses illustrate the dynamic interplay between NELF and the nucleosome to establish the promoter-proximal checkpoint.

## RESULTS

### The effects of NELF and TFIIS on nucleosomal transcription by RNAPII

We examined the effects of NELF and TFIIS on transcription elongation in vitro using *Sus scrofa* RNAPII, which is highly homologous to human RNAPII, and recombinant human NELF, DSIF, and TFIIS proteins (fig. S1). First, RNAPII was loaded on a DNA/RNA scaffold (fig. S2A), and the ECs formed in the absence or presence of DSIF/NELF were analyzed by an electrophoretic mobility shift assay (EMSA) (fig. S3, A and B). While the addition of DSIF yielded a complex with lower mobility than the original EC containing only RNAPII, the addition of both DSIF and NELF yielded a complex with far lower mobility, consistent with previous studies (*20*). A complete band shift was observed when a fourfold molar excess of NELF was added to the EC containing DSIF (compare lanes 2 and 3 in fig. S3B), indicating that NELF is saturated under these conditions.

**Fig. 1.**
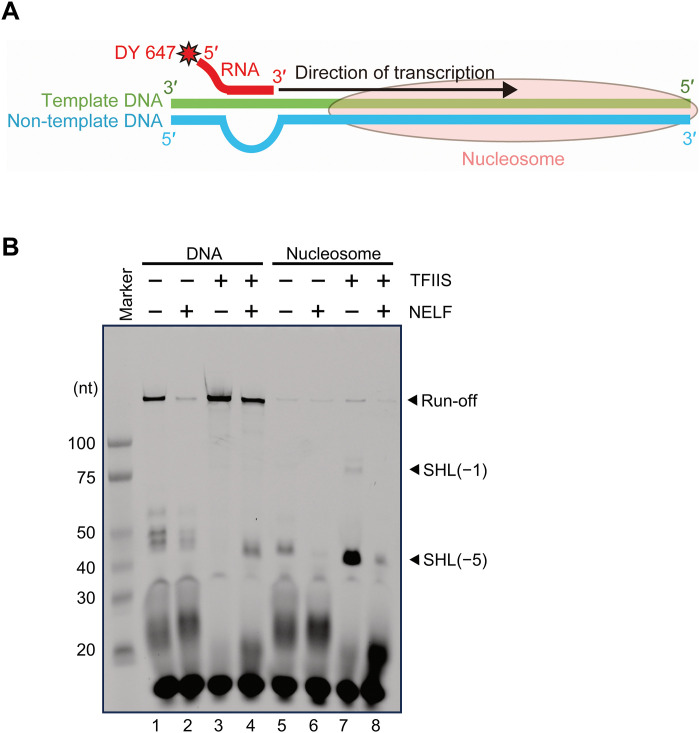
Effects of NELF and TFIIS on nucleosomal transcription by RNAPII. (**A**) Schematic representation of the nucleosomal template. The template DNA, nontemplate DNA, and fluorescently labeled primer RNA are colored green, cyan, and red, respectively. (**B**) Urea PAGE analysis of RNA transcripts generated by the transcription reactions in the presence or absence of NELF and TFIIS. DSIF is included in all lanes. The experiment was performed in duplicate (fig. S4A).

We then examined the effects of NELF and TFIIS on nucleosomal transcription. RNAPII was loaded onto the temp42/58 DNA (*21*) or nucleosomal DNA reconstituted with the temp42/58 DNA (fig. S2B), and transcription was performed in the absence or presence of these factors (Fig. 1). In the absence of TFIIS, short transcripts (less than 30 nt) accumulated on the naked DNA and the nucleosomal template (lanes 1, 2, 5, and 6 in Fig. 1B). These RNAs likely originated due to RNAPII backtracking or arrest and increased by the addition of NELF (lanes 2 and 6). TFIIS reactivates the backtracked/arrested RNAPII by stimulating the RNA cleavage activity of RNAPII (fig. S3C) (*13*, *22*–*26*). In the presence of TFIIS, the short transcripts decreased, and a large fraction of RNAPII advanced to the end of the naked DNA (lane 3 in Fig. 1B), although NELF inhibited the progression (lane 4 in Fig. 1B). On the nucleosomal template, RNAPII advanced to the nucleosome and stalled around superhelical location –5 [SHL(–5)], which is one of the strongest barrier positions within the nucleosome (lane 7 in Fig. 1B) (*27*, *28*). In addition, some RNAPII fractions passed through SHL(–5) and reached SHL(–1) or the end of the template (run-off). By contrast, the addition of NELF substantially decreased these fractions but increased the amount of short RNAs (lane 8 in Fig. 1B). The enhanced production of the short RNAs, compared to those generated from the naked DNA template (compare lanes 4 and 8), suggests that NELF and the nucleosome cooperate to enhance the transcriptional barrier.

### Structures of AEC-nuc

**Fig. 2.**
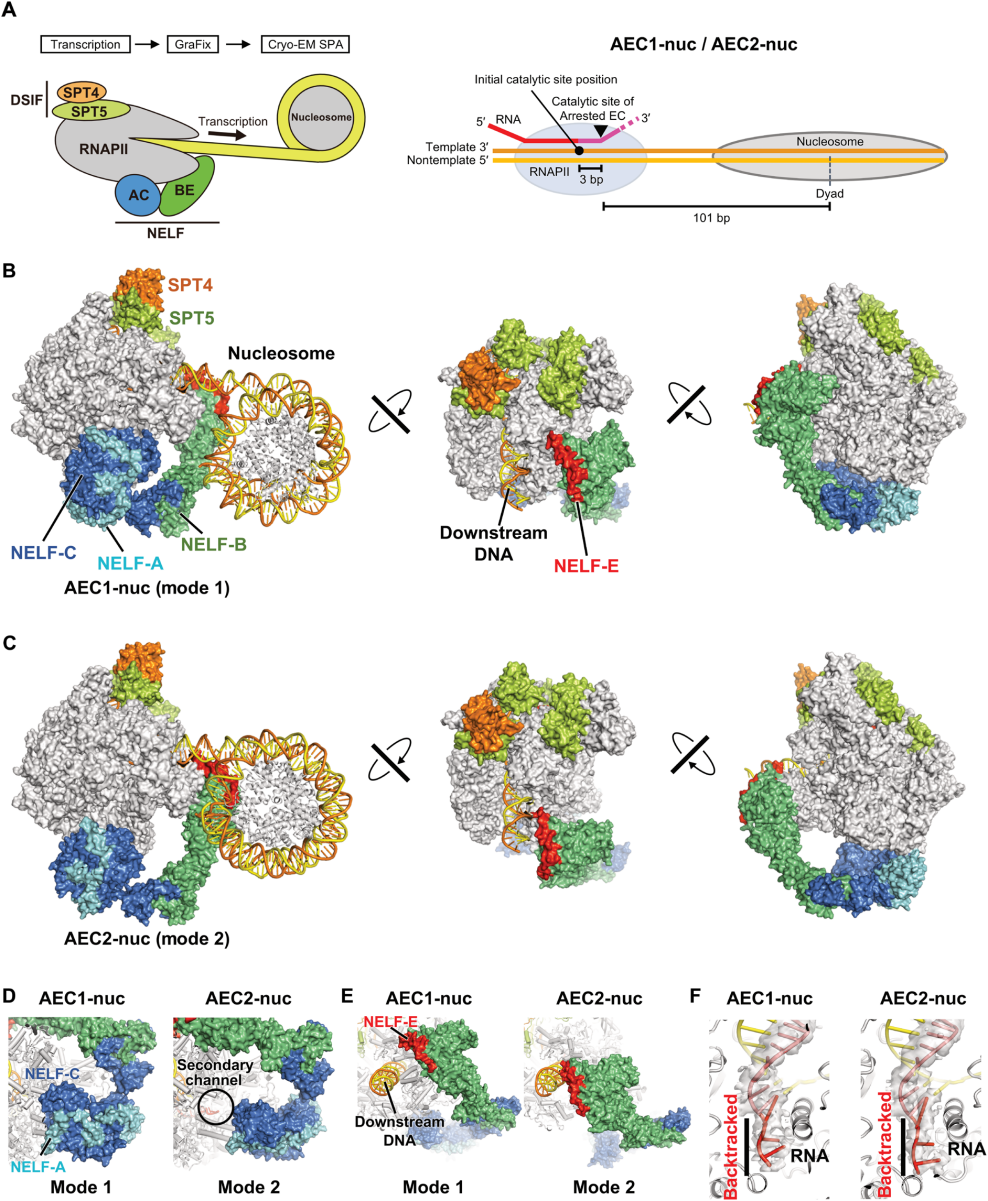
Cryo-EM structures of arrested ECs in two alternative NELF-binding modes. (**A**) Left panel shows schematic of the cryo-EM sample preparation. The right panel depicts the RNAPII position relative to the nucleosome in obtained complex structures. (**B**) Overall structure of arrested EC with NELF-binding mode 1 (AEC1-nuc) in three orientations. In the middle and right panels, the nucleosome is omitted for visibility. RNAPII, SPT4/SPT5, and NELF-A/NELF-B/NELF-C/NELF-E are shown as gray, orange/light green, and cyan/green/blue/red surfaces, respectively. Histone proteins, template/nontemplate DNA, and RNA are shown as gray, yellow/orange, and red ribbon models. (**C**) Overall structure of arrested EC with NELF-binding mode 2 (AEC2-nuc) in three orientations. (**D**) Comparison of the NELF-AC lobes between modes 1 and 2. (**E**) Comparison of the NELF-BE lobes between modes 1 and 2. (**F**) Close-up views of the RNAPII active site in AEC1-nuc and AEC2-nuc. Densities are represented by transparent gray surfaces. The backtracked parts of RNA are indicated. The structures in (B) to (F), represented as cartoons or surfaces, were prepared using PyMOL (Schrödinger; www.pymol.org).

To gain structural insights into RNAPII stalling, we performed cryo-EM analyses of the EC species stalled during transcription on the nucleosomal DNA template in the presence of NELF and DSIF, but without TFIIS. These transcription complexes were partially purified and chemically crosslinked by GraFix (*29*) and then subjected to cryo-EM single-particle analysis (Fig. 2, figs. S5 to S7, and table S1). Using three-dimensional (3D) classification, the NELF-containing structural classes were extracted (fig. S6). In these classes, RNAPII does not contact the downstream nucleosome, although NELF is close to the nucleosome. Further classification of the particles in these classes yielded two distinct types of complexes, with different NELF-binding modes to RNAPII (termed modes 1 and 2, Fig. 2, B and C, and fig. S6). This is consistent with the previous report that NELF/DSIF could bind to more than one site on RNAPII (*30*). In both modes, RNAPII is stalled at various locations on the nucleosomal template DNA. The major classes contained RNAPII with its catalytic site translocated either 1 or 3 bp downstream from the initial position, and we built models for the 3-bp translocated complexes (AEC1-nuc and AEC2-nuc). The nucleic acid densities in the RNAPII active site were of good quality to unambiguously assign the nucleotide sequence of the DNA/RNA hybrid and build the nucleic acid model (fig. S8, A and B).

In AEC1-nuc (mode 1), NELF binds RNAPII in a manner similar to the previously reported PEC structure (Fig. 2B and movie S1) (*11*). In contrast, AEC2-nuc revealed a different NELF binding mode (mode 2, Fig. 2C and movie S2). NELF can be divided into two lobes, the NELF-BE and NELF-AC lobes, which are located close to the RPB5 and RPB8 subunits of RNAPII, respectively. In mode 1, the NELF-AC lobe blocks the RNAPII secondary channel, which is the TFIIS binding site (Fig. 2D). In mode 2, the NELF-AC lobe is farther away from the secondary channel, exposing the TFIIS-binding site (Fig. 2D). Furthermore, the NELF-BE lobe in mode 2 is reoriented toward the downstream DNA, forming extensive contacts with the RNAPII RPB5 subunit (Fig. 2E). This change causes a ~35-Å shift of the N-terminal α helix of NELF-E (α1), the only resolved part of the NELF-E subunit, which forms direct contacts between the α1 helix and the downstream DNA backbone (Fig. 2E). As the classification identified an intermediary structural state between modes 1 and 2 (“mode 1.5,” fig. S9), the transition from mode 1 to mode 2 could occur through the NELF-AC lobe relocation, followed by the NELF-BE reorientation (movie S3). Collectively, NELF binding transitions between two alternative modes, and NELF in mode 2 is compatible with TFIIS binding and directly contacts the downstream DNA through the NELF-E helix.

Another prominent feature of the current arrested ECs with a nucleosome (AEC-nuc) structures is that RNAPII is in a backtracked/arrested state. In the previously reported PEC structures, the RNAPII active site contained a “tilted” DNA/RNA hybrid, which is incompatible with RNA elongation (*11*). In contrast, in AEC1-nuc and AEC2-nuc, the conformation of the DNA/RNA hybrid is “straight,” and similar to that in an active EC (fig. S8, A to D) (*31*). The cryo-EM densities reveal 3 to 4 nt of RNA extruded into the RNAPII secondary channel (Fig. 2F and fig. S8, E and F). Thus, the current AEC-nuc structures were generated by RNAPII backtracking by a certain distance on the nucleosomal template DNA. This is consistent with the accumulation of short RNAs in the absence of TFIIS (Fig. 1B). While RNAPII appears to have backtracked by ~5 nt (Fig. 1B), the 3′ end of the RNA may be disordered in the AEC-nuc structures. As the NELF-BE lobe is close to the nucleosome, steric hindrance between NELF and the nucleosome could cause RNAPII backtracking, as discussed below.

### The NELF-DNA contact in mode 2 is critical for RNAPII pausing

**Fig. 3.**
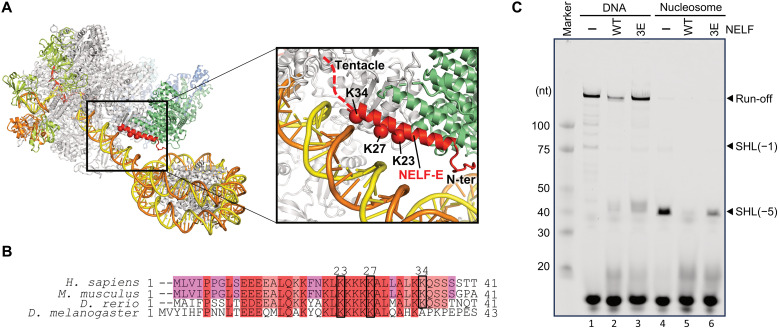
The contact between NELF-E and the downstream DNA is critical for pausing. (**A**) Overall structure of the AEC2-nuc complex (left) and close-up view of the interface between the NELF-E basic helix and the downstream DNA. The Cβ atoms of K23, K27, and K34 are shown as sphere models. The structures, depicted as cartoons, were prepared using PyMOL. (**B**) Sequence alignment of the N-terminal regions of NELF-E. (**C**) Transcription assay with the mutant NELF, in which the three Lys residues are replaced by Glu (3E). Urea PAGE analysis of the transcription products is shown. The experiment was performed in triplicate (fig. S4B).

The AEC2-nuc structure revealed the direct contact of the N-terminal α1 helix of NELF-E with the downstream DNA. The NELF-E α1 helix is a Lys-rich helix, and the side chains of Lys^23^, Lys^27^, and Lys^34^ potentially contact the DNA phosphate groups (Fig. 3, A and B). To assess the significance of these contacts, we prepared a mutant NELF, in which all three Lys residues were replaced by Glu (K23E-K27E-K34E, 3E) and performed transcription assays (Fig. 3C and fig. S3D). The mutant NELF exists as a NELF-A/NELF-B/NELF-C/NELF-E complex with a molecular mass comparable to the wild-type NELF complex, as estimated by a mass photometry analysis (fig. S1, A and C). Similar to the wild-type NELF, the mutant NELF forms an RNAPII-DNA/RNA-NELF-DSIF complex, as shown by the EMSA analysis (fig. S3B). However, while wild-type NELF inhibits transcription progression in the presence of TFIIS on a naked DNA template, the 3E mutation attenuated this inhibitory effect (lanes 2 and 3 in Fig. 3). This trend is also observed on a short DNA/RNA scaffold, and the 3E mutant does not inhibit transcription as efficiently as wild-type NELF, even at a 10-fold excess concentration (fig. S3D). Thus, the NELF-binding mode 2 is involved in RNAPII pausing/stalling, where the downstream DNA contacts by the NELF-E basic helix play a critical role.

We then examined the effect of NELF on transcription on a nucleosomal DNA. As compared with the conditions without NELF, only limited amounts of RNAPII reached the nucleosome in the presence of wild-type NELF, suggesting that the nucleosome and NELF cooperatively prevent the RNAPII progression (Fig. 3C, lane 5). In AEC2-nuc, RNAPII does not reach the downstream nucleosome, but the NELF-BE lobe is close to the nucleosome (Fig. 2C). RNAPII progression causes rotation of the nucleosome around the downstream DNA axis (*32*). An approximately 6-bp progression of the EC from the priming site would cause collisions between the NELF-E helix and the nucleosome, and this steric hindrance could be the source of the NELF-nucleosome–mediated inhibition (fig. S10). The NELF-E mutation (3E) partially alleviated the inhibition, albeit to a limited extent compared to that in the absence of NELF (Fig. 3C, lane 6). This suggests that, in addition to the direct contact between the NELF-E basic helix and DNA, steric hindrance between NELF and the nucleosome contributes to the inhibition of EC progression.

### Structure of PEC bound with TFIIS and a nucleosome (PEC2-nuc)

**Fig. 4.**
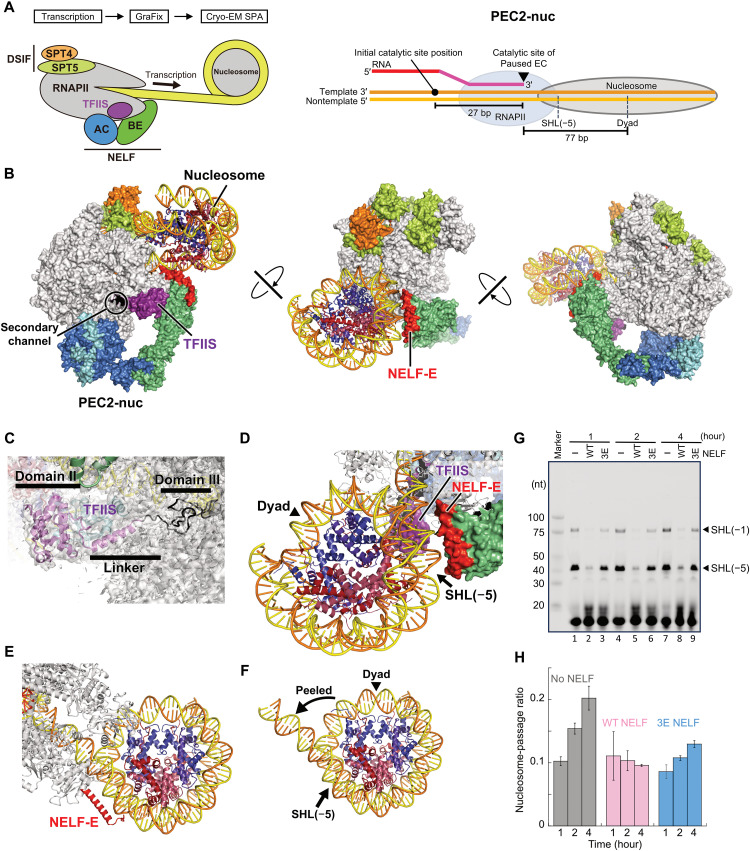
Cryo-EM structure of PEC with a nucleosome (PEC2-nuc). (**A**) The left panel shows schematic of the cryo-EM sample preparation. The right panel depicts the RNAPII position relative to the nucleosome in the obtained complex structure. (**B**) Overall structure of PEC2-nuc in three orientations. RNAPII, NELF, and DSIF are colored as in Fig. 2C. TFIIS is represented as a violet surface. (**C**) TFIIS binding site. The density is shown as a transparent gray surface. Although the density of the TFIIS domain III is poor, its model is shown for visibility. (**D** and **E**) Close-up views of the nucleosome and its interactions with NELF and RNAPII. (**F**) Partially unwrapped nucleosome in the complex. (**G**) Time course of transcription with NELF (the wild type and 3E mutant). Urea PAGE analysis of the transcription products is shown. The experiment was performed in triplicate (fig. S4C). (**H**) Nucleosome-passage ratio. Band intensities in (G) were quantitated, and the calculated nucleosome-passage ratios (intensity [SHL(–1)]/{intensity [SHL(–1)] + intensity [SHL(–5)]}) were plotted. The mean values ± SD from three independent experiments are shown (*n* = 3 technical replicates). The structures in (A) to (F), represented as cartoons or surfaces, were prepared using PyMOL. WT, wild type.

Subsequently, we performed RNAPII transcription on the nucleosomal DNA template in the presence of DSIF, NELF, and TFIIS and analyzed the formed complex species by cryo-EM (Fig. 4A and figs. S11 and S12). All RNAPII-containing structural classes showed the density of the RNAPII-associated TFIIS. We extracted the NELF-containing classes from these classes. In some of the NELF-containing classes, RNAPII had advanced to collide with the downstream nucleosome, and we reconstructed the PEC structure containing RNAPII, DSIF, NELF, TFIIS, and the nucleosome (PEC2-nuc; Fig. 4B and movie S4). In this structure, NELF binds RNAPII in mode 2, simultaneously with TFIIS.

TFIIS binds the rim of the RNAPII secondary channel, in a manner similar to that observed previously (Fig. 4C) (*24*, *33*). The TFIIS domain II is bound to the jaw domain of the RNAPII RPB1 subunit. The interdomain linker connecting domains II and III extends along the RPB1 funnel helices to the RNAPII secondary channel. The domain III density is weak, probably because of its flexibility. There is no apparent change in the RNAPII conformation, as compared with the AEC2-nuc complex lacking TFIIS (fig. S13). In the RNAPII active site, the DNA/RNA hybrid is primarily in the post-translocation state, consistent with the RNA cleavage stimulating function of TFIIS (fig. S14). There is no apparent contact or steric clash between TFIIS and NELF.

After stripping ~20 bp of the nucleosomal DNA from the histone surfaces, RNAPII is stalled within the downstream nucleosome (Fig. 4, D to F). The leading edge of RNAPII is positioned around SHL (–5) of the nucleosome, consistent with the previous in vivo observation (*16*). In addition, the RNAPII RPB1 jaw (1264 to 1279) directly contacts the exposed N-terminal helix of histone H3 (fig. S15, A and B). The RNAPII-nucleosome interaction is similar to that observed in the yeast *Komagataella pastoris* RNAPII stalled at SHL(–5) within a nucleosome (fig. S15C) (*27*), suggesting the importance of the nucleosomal barrier in RNAPII pausing. In PEC2-nuc, the NELF-E basic helix directly contacts the nucleosomal DNA (Fig. 4, D and E). RNAPII progression causes rotation of the nucleosome around the downstream DNA axis (*32*), and only about a 2-bp progression from the observed stall site would cause clashes between the NELF-E basic helix and the nucleosome (fig. S16). This suggests that the NELF-nucleosome interaction enhances the transcriptional barrier.

To assess the effects of NELF and the nucleosome on the nucleosome passage by RNAPII, we performed a time-course analysis of transcription on the nucleosomal template in the absence or presence of NELF (wild type or 3E mutant) (Fig. 4G). In this experiment, we replaced adenosine 5′-triphosphate (ATP), one of the four nucleoside triphosphate (NTP) substrates, with 3′-deoxy ATP. As the temp42 template has the first T tract at 42-bp downstream from the nucleosome entry (fig. S2B), the RNAPII that overcame the SHL(–5) barrier would accumulate around SHL(–1) of the nucleosome. The nucleosome-passage ratio is defined as the fraction of RNAPII that reached SHL(–1) divided by the fraction of RNAPII that reached the nucleosome {band intensities around SHL(–1)/band intensities of [SHL(–5) + SHL(–1)]}. While the nucleosome-passage ratio increased with time in the absence of NELF, it remained almost unchanged in the presence of NELF (Fig. 4, G and H). Thus, the PEC2-nuc complex formed near the entry of the nucleosome strongly restricts RNAPII progression downstream beyond SHL(–5). The 3E mutation of NELF-E partly alleviated this restriction (Fig. 4, G and H), suggesting the importance of the direct NELF-nucleosome interactions/conflicts for this restriction.

## DISCUSSION

**Fig. 5.**
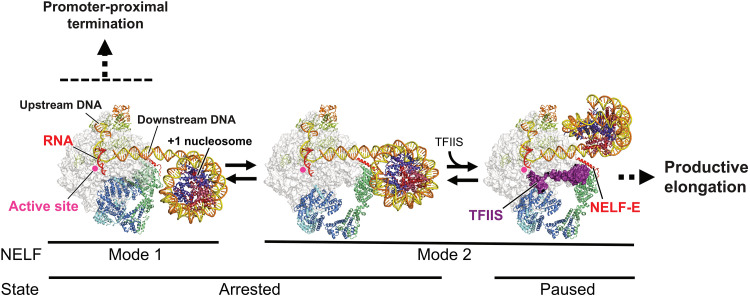
Structural model of promoter-proximal pausing. NELF and DSIF maintain a stalled EC. The NELF-binding mode is in equilibrium between mode 1 and mode 2. The interaction/conflict between NELF and the +1 nucleosome causes EC backtracking to form arrested ECs (left and middle). When NELF assumes mode 2 (middle), TFIIS can bind RNAPII to reactivate the arrested EC via its RNA-cleavage stimulating function. The EC then advances to the +1 nucleosome and eventually forms a PEC within a partially unwrapped nucleosome (right). NELF and the nucleosome cooperate to strongly restrict the EC progression further downstream, until pause release for productive elongation. In this figure, the DNA/RNA structures are superimposed on the protein structures to indicate their paths. The structures, represented as cartoons or surfaces, were prepared using PyMOL.

Emerging evidence suggests that the +1 nucleosome plays a critical role in promoter-proximal pausing of RNAPII (*14*–*19*). In the present study, we determined the cryo-EM structures of mammalian ECs containing RNAPII, NELF, DSIF, and/or TFIIS stalled on a nucleosomal DNA (Fig. 5). In the arrested EC structures (AEC1-nuc and AEC2-nuc) formed in the absence of TFIIS, RNAPII is arrested before reaching the nucleosome, although NELF is close to and interacts with the nucleosome. These structures revealed that NELF adopts two alternative binding modes to RNAPII: mode 1 and mode 2. In the presence of TFIIS, the EC binds both TFIIS and NELF in mode 2, and advances to the nucleosome to form a PEC (PEC2-nuc) within the nucleosome. These structures provide a dynamic view of promoter-proximal pausing in the context of chromatin (Fig. 5).

In AEC1-nuc and AEC2-nuc (Fig. 5, left and middle), RNAPII is backtracked with the RNA 3′ end extruding into the RNAPII secondary channel. The previously reported PEC structure revealed a “tilted” DNA/RNA hybrid within the RNAPII active site, indicative of an inactive state (*11*). Recently, these authors reported another PEC structure, in which RNAPII is in a post-translocation state with a regular, “straight” DNA/RNA hybrid competent for RNA elongation (*34*). However, RNAPII is prone to backtracking in early elongation (*35*), and the existence of these backtracked/arrested ECs was expected. The current AEC-nuc structures likely reflect these states. While the DNA sequence context is critical for RNAPII backtracking, the presence of the downstream nucleosome may be another cause of backtracking (*33*). In AEC1-nuc and AEC2-nuc, the RNAPII active site stalls ~25-bp upstream of the nucleosome entry point. However, the NELF-BE lobe forms the downstream edge of the EC, and the interactions/conflicts between NELF and the nucleosome could cause backtracking.

Our current structures revealed two alternative modes of NELF binding to RNAPII (Fig. 5), which are consistent with the previous biochemical study (*30*) and recent structural studies (*34*, *36*). In mode 2 (AEC2-nuc), the NELF-AC lobe, which blocks the RNAPII secondary channel in mode 1 (AEC1-nuc), is repositioned to allow TFIIS to access the secondary channel. In PEC2-nuc, TFIIS is indeed bound to the exposed binding site. These modes explain the previous observations that TFIIS colocalizes with the promoter proximally paused/stalled RNAPII at the uninduced *Drosophila hsp70* promoter (*37*) and that TFIIS is required for the reactivation of the backtracked/arrested RNAPII for the pause release (*37*, *38*).

Transcription assays revealed that the inhibitory effect of NELF is maintained even in the presence of TFIIS (Figs. 1B and 3C). This suggests that the NELF-binding mode 2 is involved in an alternative paused state, but not a fully active state. As compared with mode 1, the mode 2 structures (AEC2-nuc and PEC2-nuc) showed large repositioning of the NELF-BE lobe, which is critical for the pausing (Fig. 5). First, the basic helix of the NELF-E subunit directly contacts the downstream DNA, and this interaction is essential for transcription inhibition. The contacts between SPT5 (one of the DSIF subunits) and DNA/RNA are reportedly crucial for the RNAPII pausing (*39*). DNA/RNA contacts by multiple NELF and DSIF domains may cooperatively lower the RNAPII processivity, and the DNA contact by NELF-E should be particularly important when TFIIS is bound to RNAPII and NELF assumes mode 2. Second, transcription inhibition by NELF is stronger on a nucleosomal DNA template than on naked DNA. As described, the NELF-BE lobe forms the downstream edge of the EC, causing steric conflicts with the nucleosome before (AEC2-nuc) and after (PEC2-nuc) RNAPII reaches the nucleosome. Thus, the physical interactions/conflicts between NELF and the nucleosome seem to generate barriers against transcription over a wide region, from the upstream side to the inside of the +1 nucleosome. These strong barriers would cause RNAPII backtracking (*16*, *33*, *35*), and the iteration of the RNAPII backtracking, reactivation by TFIIS, and subsequent RNAPII progression to the barrier site may maintain the pausing, accounting for the previously described paused or “poised” RNAPII (*40*, *41*).

In the presence of TFIIS, EC advances to form a PEC with the downstream nucleosome (PEC2-nuc) (Fig. 5, right). In this structure, RNAPII is stalled within a partially unwrapped nucleosome, with its leading edge at SHL(–5), one of the strongest barrier sites against RNAPII progression (*27*, *32*). NELF reinforces the barrier through direct interactions between NELF-E and the nucleosome. Thereby, NELF and the nucleosome seem to cooperatively restrict RNAPII progression beyond SHL(–5) of the nucleosome. This explains the previous observation that the acute depletion of NELF causes a downstream shift of RNAPII beyond the first pause site around SHL(–5) to the second pause site within the +1 nucleosome (*19*, *42*).

The promoter-proximal pausing is believed to serve as a checkpoint before the RNAPII pause release into the gene body. To overcome the +1 nucleosome, a PEC needs to transition into a productive EC by exchanging NELF with elongation factors such as SPT6 and PAF1C, and this is regulated by P-TEFb and other factors specific to developmental and stimulus-responsive gene regulation (*1*–*4*, *31*, *43*). Our current structures represent a solid framework for these events and regulations. In addition, RNAPII in the promoter-proximal region is prone to premature termination (*44*–*47*), and our structures also provide insights into how the Integrator complex detects a stalled EC that needs to be terminated. NELF and DSIF serve as interfaces with the Integrator complex, where the NELF-B subunit interacts with the Integrator INTS6 subunit (*48*–*50*). As NELF-binding mode 2 appears to be incompatible with INTS6 binding, the mode 1 complexes, such as AEC1-nuc, could be preferred targets for termination (fig. S17).

## MATERIALS AND METHODS

### Protein expression and purification

*S. scrofa* RNAPII was purified from *S. scrofa* thymus (purchased from Tokyo Shibaura Zouki Company Limited) as described previously (*51*). Briefly, the *S. scrofa* thymus was pulverized in a 2-liter blender (Waring) in buffer A [50 mM Tris-HCl (pH 7.9), 1 mM EDTA, 10 μM ZnCl_2_, 10% glycerol, 1 mM dithiothreitol (DTT), 1 mM phenylmethylsulfonyl fluoride, 1 mM benzamidine, 0.6 μM leupeptin, and 2 μM pepstatin A]. The thymus suspension was filtered through two layers of Miracloth (Millipore). The supernatant was precipitated with 0.1% polyethylenimine, and this precipitate was washed with buffer A. The RNAPII fraction was extracted from the precipitate with buffer A supplemented with 150 to 200 mM ammonium sulfate. RNAPII was purified by anion-exchange column chromatography on a Q-Sepharose column (Cytiva), ammonium sulfate precipitation, and size exclusion chromatography on a Superose 6 Increase column (Cytiva) equilibrated with buffer B [5 mM Hepes-NaOH (pH 7.3), 150 mM NaCl, 10 μM ZnCl_2_, and 10 mM DTT]. Peak fractions containing RNAPII were collected and stored as a 10% glycerol stock at −80°C. For biochemical analyses, RNAPII was further purified by affinity purification using an anti-RNAPII antibody [8WG16] (Santa Cruz Biotechnology) (fig. S1) (*52*).

For the expression of human DSIF (the SPT4/SPT5 complex), the genes encoding the N-terminally His-tagged SPT4 and the C-terminally Strep-tagged SPT5 were cloned into the pETDuet-1 vector (Novagen). SPT4 and SPT5 were expressed in the *Escherichia coli* strain Rosetta 2 (DE3, BL21). The cells were resuspended in buffer C [20 mM Tris-HCl (pH 8.0) 500 mM NaCl, 0.1 mM phenylmethylsulfonyl fluoride, 1 mM benzamidine, 10 mM 2-mercaptoethanol, and 1 mM EDTA] supplemented with 20 mM imidazole, sonicated, and then cleared by centrifugation. The supernatant was applied to a Ni Sepharose 6 Fast Flow column (Cytiva), which was washed extensively with buffer C supplemented with 20 mM imidazole. The sample was eluted using buffer C supplemented with 500 mM imidazole. Subsequently, tobacco etch virus protease (4 mg/ml) was added, and the mixture was dialyzed against buffer D [20 mM Tris-HCl (pH 8.0), 150 mM NaCl, and 1 mM DTT] supplemented with 20 mM imidazole. To remove the cleaved His-tag and the noncleaved protein, the sample was again applied to a Ni Sepharose 6 Fast Flow column, and the flow-through fraction was collected. The protein was further purified by anion-exchange chromatography using a Resource Q column, using a linear gradient from buffer D to buffer E [20 mM Tris-HCl (pH 8.0), 1 M NaCl, and 1 mM DTT], followed by gel-filtration column chromatography on a Superose 6 Increase column (Cytiva) equilibrated with buffer F [20 mM Hepes-NaOH (pH 7.5), 150 mM NaCl, and 1 mM DTT]. The purified protein was concentrated with an Amicon Ultra centrifugal filter unit (Merck).

For the preparation of the human NELF complex, the genes encoding NELF-A, NELF-B, the C-terminally His6-tagged NELF-C, and the N-terminally FLAG-tagged NELF-E subunits were tandemly cloned into the pACEBac1 vector (Geneva Biotech). The 3E mutant (K23E, K27E, and K34E) was constructed using site-directed mutagenesis. Both the wild-type and 3E mutant NELF complexes were expressed in High Five cells. The cells were resuspended in buffer C supplemented with 10 μM ZnCl_2_ and 20 mM imidazole, disrupted by sonication, and then cleared by centrifugation and filtration. The clarified lysate was applied to a Ni Sepharose 6 Fast Flow column (Cytiva), which was washed extensively with buffer C supplemented with 10 μM ZnCl_2_ and 20 mM imidazole. Lambda protein phosphatase was added (~0.1 mg/ml) to dephosphorylate NELF. The sample was dialyzed against buffer G [20 mM Tris-HCl (pH 8.0), 150 mM NaCl, 1 mM DTT, 10 μM ZnCl_2_, and 1 mM MnCl_2_] and then further purified by anion-exchange chromatography on a Resource Q column (Cytiva) using a linear gradient from buffer D supplemented with 10 μM ZnCl_2_ to buffer E supplemented with 10 μM ZnCl_2_, followed by gel-filtration column chromatography on a Superose 6 Increase column (Cytiva) equilibrated with buffer H [20 mM Hepes-NaOH (pH 8.0), 150 mM NaCl, 10 μM ZnCl_2_, and 1 mM DTT]. Fractions were collected and then concentrated using an Amicon Ultra filter.

Human TFIIS was prepared as described previously (*51*). In brief, the human TFIIS gene was cloned into the pET15b vector (Novagen). The TFIIS mutants deficient in RNA-cleavage stimulating activity were created by mutating or deleting essential acidic residues [D282A and E283A (aa); deletion of D282 and E283 (Δ)]. The wild-type and mutant TFIIS proteins were expressed as His6-tagged proteins in *E. coli.* The cells were resuspended in buffer C supplemented with 20 mM imidazole, sonicated, and then cleared by centrifugation. The supernatant was applied to a Ni Sepharose 6 Fast Flow column (Cytiva), which was washed extensively with buffer C supplemented with 20 mM imidazole, and then eluted by cleavage of the His6-tag using human rhinovirus 3C protease. TFIIS was further purified by Resource S cation-exchange column chromatography (Cytiva) using a linear gradient from buffer D to buffer E, followed by gel-filtration column chromatography on a Superdex 200 column (Cytiva) equilibrated with buffer I [5 mM Hepes-NaOH (pH 7.4), 150 mM NaCl, 10 μM ZnCl_2_, and 1 mM DTT].

For the nucleosome preparation, human histones H2A, H2B, H3.3, and H4 were purified as described previously (fig. S2B) (*53*). Briefly, the His6-tagged histone proteins were expressed in *E. coli* and purified by Ni-NTA affinity column chromatography (QIAGEN). After His6-tag removal by thrombin protease, the histone proteins were further purified by Mono S cation-exchange column chromatography (Cytiva). The purified histone proteins were lyophilized, and the resulting histone powders were stored at 4°C. The histone octamer was reconstituted as described previously (*53*). Briefly, the four histone powders were dissolved together in denaturing buffer, and the histone octamer was reconstituted by dialysis against refolding buffer. The reconstituted histone octamer was purified by Superdex 200 gel filtration column chromatography (Cytiva).

### Reconstitution of the nucleosome

The temp42/58 DNA fragment was designed and purified as described previously (*27*). The temp42 DNA fragment was designed as below and purified as described previously (*27*). The nucleosome was reconstituted with the DNA fragment and the histone octamer by the salt dialysis method (*53*). The reconstituted nucleosome was ligated to the DNA fragment containing the 9-bp mismatched region by T4 DNA ligase (NIPPON GENE). The resulting nucleosome containing a linker with a mismatched region was further purified by nondenaturing PAGE using a Prep Cell apparatus. The DNA sequence of temp42 is as follows: nontemplate strand (5′-TGGCCGTTTTCGTTGTTTTTTTCTGTCTCGTGCCTGGTGTCTTGGGTGTAAAACCCTTGGCGGTTAAAACGCGGGGGACAGCGCGTACGTGCGTTTAAGCGGTGCTAGAGCTGTCTACGACCAATTGAGCGGCCTCGGCACCGGGATTCTGAT-3′); and template strand (5′-ATCAGAATCCCGGTGCCGAGGCCGCTCAATTGGTCGTAGACAGCTCTAGCACCGCTTAAACGCACGTACGCGCTGTCCCCCGCGTTTTAACCGCCAAGGGTTTTACACCCAAGACACCAGGCACGAGACAGAAAAAAACAACGAAAACGGCCACCA-3′).

### Mass photometry analysis

The Refeyn TwoMP mass photometer was first calibrated using 20 nM standard marker proteins: bovine serum albumin (67 kDa), immunoglobulin G (150 kDa), and thyroglobulin (660 kDa). Wild-type NELF and the 3E mutant were diluted to 10 nM with buffer D and measured. The Refeyn Acquire software was used for data acquisition, and the Refeyn Discover software was used for analysis.

### Electrophoretic mobility shift assay

The DNA/RNA scaffold (fig. S2A) was annealed by incubating a mixture of 5 μM Alexa Fluor 647–labeled primer RNA (Dharmacon), 5 μM template DNA, and 5 μM nontemplate DNA at 70°C for 5 min, followed by cooling to room temperature. The 0.26 μM RNAPII was first incubated with an equal amount of the DNA/RNA scaffold and 3 U of ribonuclease (RNase) inhibitor (TAKARA) at 30°C for 10 min. This mixture was then mixed with DSIF and/or NELF, as indicated in fig. S3B, in 5 μl of buffer J [20 mM Hepes-NaOH (pH 7.5), 5 mM MgCl_2_, and 5% (v/v) glycerol]. Final concentrations of RNAPII, DSIF, and NELF were 0.1, 0.4, and 0.4 μM, respectively. The final salt concentrations are 75 mM NaCl, 50 mM KOAc, 0.33 μM Zn(OAc)_2_, 5 mM MgCl_2_, and 4 μM ZnCl_2_. Each solution was incubated for 30 min at 4°C, and then the complex species were separated by native–polyacrylamide gel electrophoresis (PAGE) on a NuPAGE 3 to 8% Tris-acetate gel (Invitrogen). Alexa Fluor 647 fluorescence signals were detected with an ImageQuant LAS4000 image analyzer (Cytiva).

### Transcription assay

The transcription reaction on the DNA/RNA scaffold (fig. S2A) was performed in 5 μl of reaction buffer J, containing 0.1 μM RNAPII and 0.1 μM DNA/RNA scaffold, with various combinations of NELF (0.4 or 1 μM), DSIF (0.4 μM), and TFIIS (0.1 μM). First, 0.26 μM RNAPII was incubated with an equal amount of the DNA/RNA scaffold and 3 U of RNase inhibitor (TAKARA) at 30°C for 10 min. This mixture was then mixed with DSIF, TFIIS, and/or NELF. The reaction was initiated by adding ^1^/_10_ volume of 10× buffer J containing the NTP mix solution [0.4 mM uridine 5′-triphosphate (UTP), 0.4 mM cytidine 5′-triphosphate (CTP), 0.4 mM guanosine 5′-triphosphate (GTP), and 0.4 mM ATP, final concentrations], and the mixtures were incubated for 20 min at 30°C. The final salt concentrations in the reaction are 75 mM NaCl, 50 mM KOAc, 0.33 μM Zn(OAc)_2_, 5 mM MgCl_2_, and 4 μM ZnCl_2_. After the incubation, the reaction was quenched by adding 1 μl of stop solution (100 mM HEPES-KOH (pH 7.4), 1% SDS, 100 mM EDTA, 113 mg/mL Proteinase K), and was further incubated for 30 min at 55°C. Then, 6 μl of 2× formamide dye [93% (w/v) formamide, 5 μM nontemplate DNA, 0.1% (w/v) Orange G (Merck), and 9.5 mM EDTA (pH 8.0)] was added to the samples, and the samples were heated for 10 min at 85°C. The resulting RNAs were analyzed by denaturing-PAGE on 10% Novex TBE-Urea gels (Invitrogen). Alexa Fluor 647 fluorescence signals were detected with an LAS4000 image analyzer (Cytiva). Prestained Small RNA Plus (Biodynamics Laboratory) markers were used for the gel electrophoresis.

The transcription assays on the nucleosomal template (fig. S2B) were performed as follows. For the experiment shown in Fig. 1B, the indicated combinations of 0.1 μM RNAPII, 0.6 μM DSIF, 0.6 μM NELF, 0.1 μM TFIIS, 0.1 μM DY647 fluorescently labeled RNA primer, and 0.1 μM temp42/58 nucleosome or DNA were combined in a 9 μl of reaction mixture and incubated for 30 min at 37°C to assemble the complex. Then, the transcription reaction was initiated by adding 1 μl of the NTP mix solution, and the mixtures were incubated for 30 min at 37°C. The resulting 10-μl reaction solution contained 34 mM Hepes-NaOH (pH 7.5), 68 mM KOAc, 2 μM ZnCl_2_, 0.05 mM TCEP, 13% glycerol, 33 mM NaCl, 0.27 mM DTT, 5 mM MgCl_2_, 0.4 mM UTP, 0.4 mM CTP, 0.4 mM GTP, and 0.4 mM ATP.

For the experiment shown in Fig. 3C, the indicated combinations of 0.1 μM RNAPII, 0.6 μM DSIF, 0.6 μM NELF, 0.1 μM TFIIS, 0.1 μM DY647 fluorescently labeled RNA primer, and 0.1 μM temp42/58 nucleosome or DNA were combined in a 13.5 μl of reaction mixture and incubated for 30 min at 37°C to assemble the complex. Then, the transcription reaction was initiated by adding 1.5 μl of the NTP mix solution, and the mixtures were incubated for 30 min at 37°C. The resulting 15 μl of reaction solution contained 34 mM Hepes-NaOH (pH 7.5), 54 mM KOAc, 2.6 μM ZnCl_2_, 0.04 mM TCEP, 13% glycerol, 43 mM NaCl, 0.37 mM DTT, 5 mM MgCl_2_, 0.4 mM UTP, 0.4 mM CTP, 0.4 mM GTP, and 0.4 mM ATP.

For the experiment shown in Fig. 4G, the indicated combinations of 0.1 μM RNAPII, 0.6 μM DSIF, 0.6 μM NELF, 0.2 μM TFIIS, 0.1 μM DY647 fluorescently labeled RNA primer, and 0.08 μM temp42 nucleosome were combined in a 13.5 μl of reaction mixture and incubated for 30 min at 37°C to assemble the complex. Then, the transcription reaction was initiated by adding 1.5 μl of the NTP mix solution, and the mixtures were incubated for 1, 2, and 4 hours at 37°C. The resulting 15 μl of reaction solution contained 34 mM Hepes-NaOH (pH 7.5), 70 mM KOAc, 2 μM ZnCl_2_, 0.05 mM TCEP, 13% glycerol, 33 mM NaCl, 0.27 mM DTT, 5 mM MgCl_2_, 0.4 mM UTP, 0.4 mM CTP, 0.4 mM GTP, and 0.4 mM 3′-dATP.

After the incubation, a 2-μl aliquot of the reaction mixture was quenched by adding 1 μl of stop solution [100 mM Tris-HCl (pH 8.0), 4 M urea, 150 mM EDTA, and Proteinase K (1 mg/ml)] and further incubated at room temperature for 5 min. The resulting samples were mixed with Hi-Di formamide (Thermo Fisher Scientific) and heated at 95°C for 5 min to denature the elongated RNA. The fluorescently labeled RNA was analyzed by 10% denaturing urea PAGE. The DY647 fluorescence signals were detected through the glass plate using an Amersham Typhoon imager (Cytiva), with linear contrast enhancement. Blue-colored RNA markers (Biodynamics Laboratory) were used. The band intensities at SHL(–1) and SHL(–5) in Fig. 4G were quantitated by ImageJ (*54*), and the nucleosome-passage ratios (intensity [SHL(–1)]/{intensity [SHL(–1)] + intensity [SHL(−5)]}) were calculated and plotted (Fig. 4H). The mean values ± SD from three independent experiments are shown.

### Preparation of EC-nucleosome complexes for cryo-EM

For the RNAPII-DSIF-NELF-nucleosome complex without TFIIS, the transcription reaction was conducted in two steps. In the first reaction, RNAPII, DSIF, NELF, DY647 fluorescently labeled RNA primer, and the nucleosome template were mixed in 918 μl of reaction solution containing UTP, GTP, and 3′-dATP and incubated for 10 min at 37°C. After the incubation, Cdk7/Cyclin H/MAT1 (Millipore) and CTP were added. The resulting mixture—containing 0.2 μM RNAPII, 0.6 μM DSIF, 0.6 μM NELF, 0.1 μM DY647 fluorescently labeled RNA primer, 0.6 μM Cdk7/Cyclin H/MAT1, and 0.08 μM nucleosome—was incubated for 3 hours at 37°C in 1080 μl of reaction buffer, containing 0.4 mM CTP, 0.4 mM GTP, 0.4 mM 3′-dATP, and 0.4 mM UTP. To stabilize the RNAPII-DSIF-NELF-nucleosome complex, the resulting sample was fractionated by the GraFix method (*29*). A sucrose gradient was prepared with low buffer [20 mM Hepes-NaOH (pH 7.5), 20 mM NaCl, 0.2 μM Zn(OAc)_2_, 0.1 mM TCEP-HCl, and 10% (w/v) sucrose] and high buffer [20 mM Hepes-NaOH (pH 7.5), 20 mM NaCl, 0.2 μM Zn(OAc)_2_, 0.1 mM TCEP-HCl, 25% (w/v) sucrose, and 0.1% glutaraldehyde] using a Gradient Master instrument (SKB). Centrifugation was performed for 16 hours at 27,000 rpm and 4°C using an SW41 rotor (Beckman Coulter). After centrifugation, the fractions containing the RNAPII-DSIF-NELF-nucleosome complexes were collected and dialyzed against 20 mM Hepes-NaOH (pH 7.5) buffer, containing 20 mM NaCl, 0.2 μM Zn(OAc)_2_, and 0.1 mM TCEP-HCl. The resulting samples were then concentrated with an Amicon Ultra 100 K centrifugal filter unit (Millipore).

For the RNAPII-DSIF-NELF-TFIIS-nucleosome complex, the transcription reaction was conducted in the presence of 0.2 μM TFIIS. The following GraFix, buffer exchange, and concentration procedures were performed in the same way.

For vitrification, the samples were supplemented with 0.0025% Tween 20 to improve the particle orientation distribution before the vitrification and then applied to copper Quantifoil grids (R1.2/1.3, 200 mesh; Quantifoil Micro Tools), which were glow-discharged for 2 min before use with a PIB-10 Ion Bombarder (Vacuum Device Inc.). The samples were blotted with grade 595 filter paper (Ted Pella). The grids were then plunge-frozen into liquid ethane using a Vitrobot Mark IV (Thermo Fisher Scientific) at 4°C and 100% humidity.

### Cryo-EM data collection and image processing

Cryo-EM images were recorded by a Krios G4 transmission electron microscope (Thermo Fisher Scientific) equipped with a BioQuantum energy filter and a K3 camera (Gatan). Data collection was conducted in several batches, resulting in a total of 43,663 micrographs for EC without TFIIS (arrested EC) and 33,103 micrographs for EC with TFIIS (PEC) (table S1). Image processing was conducted with Relion 3.1 (*55*), unless otherwise specified. Initial stages of data processing were performed batchwise, and after motion correction, the CTF parameters were estimated with Gctf (*56*). The initial particle picking was performed with Warp (*57*), and then the particles were subjected to preliminary 2D and 3D classifications. Several Topaz (*58*) networks were trained using particles belonging to notable classes, and the particles picked with these networks were combined and subjected to 2D and 3D classifications to remove bad particles. The particles were re-extracted, and then the 3D refinement, CTF refinement, and Bayesian polishing were performed using a mask around the EC (potential nucleosomes were excluded from the mask). Last, another 3D refinement was performed, which produced the starting dataset for the subsequent processing.

For the structural analysis of the arrested EC, the EC without TFIIS dataset was used (figs. S5 and S6). In the 3D classification, the particles were divided into three primary classes: EC with NELF in mode 1 (277,691 particles), EC with NELF in mode 2 (449,831 particles), and EC with no NELF binding (1,026,100 particles) (fig. S6). The EC class with NELF in mode 1 was subjected to NELF-focused classification, which divided the class into two subclasses with different NELF-AC lobe locations (modes 1 and 1.5; fig. S9). In both modes, further 3D classification divided the particles into ECs with different locations relative to the nucleosome. We focused on the classes in which the EC is closest to the nucleosome. The numbers of particles of modes 1 and 1.5 were 24,102 and 23,923, respectively. 3D refinement using a mask around the RNAPII-NELF-DSIF-nucleosome resulted in reconstructions at 3.3 Å (arrested EC in mode 1, map A) and 3.2 Å (arrested EC in mode 1.5, map B). Subsequently, focused refinements were performed individually for the RNAPII-DSIF, nucleosome-NELF-BE lobe, and NELF parts. These resulted in reconstructions at 3.5 Å (RNAPII-DSIF in mode 1, map A2), 3.0 Å (RNAPII-DSIF in mode 1.5, map B2), 7.2 Å (the nucleosome-NELF-BE lobe in mode 1, map A3), 7.8 Å (the nucleosome-NELF-BE lobe in mode 1.5, map B3), 7.8 Å (NELF in mode 1, map A4), and 3.3 Å (NELF in mode 1.5, map B4).

3D classification of the EC with NELF in mode 2 dataset revealed that the EC is stalled at various locations relative to the nucleosome. We further analyzed the major class, in which the EC is closest to the nucleosome (22,970 particles). 3D refinement was performed using a mask around RNAPII-DSIF/NELF-nucleosome, resulting in reconstruction at 3.3 Å (arrested EC in mode 2, map C). Subsequently, focused refinements were performed individually for the RNAPII-DSIF, the nucleosome-NELF-BE lobe, and NELF parts. These resulted in reconstructions at 3.1 Å (RNAPII-DSIF, map C2), 6.6 Å (nucleosome-NELF-BE lobe, map C3), and 5.3 Å (NELF, map C4).

For the structural analysis of the PEC (PEC2-nuc), the EC with TFIIS dataset was used (fig. S11). First, particles with strong NELF density were selected by a series of 3D classifications using a mask around the EC (excluding the nucleosome). In this dataset, we only observed mode-2 NELF, which was always accompanied by TFIIS. Next, particles containing downstream nucleosomes were enriched by global 3D classification, and then using the EC-subtracted particles, classes with good SHL(–5) nucleosomes were selected. The final 3D refinement using a mask around the RNAPII-NELF-DSIF-nucleosome resulted in a reconstruction at 3.4 Å (PEC-TFIIS/nucleosome, map D). Focused refinements were also performed for the RNAPII-DSIF-TFIIS, the nucleosome-NELF-BE lobe, and NELF parts, resulting in maps at 3.2 Å (RNAPII-DSIF-TFIIS, map D2), 3.9 Å (nucleosome-NELF-BE lobe, map D3), and 8.8 Å (NELF, map D4), respectively.

All masks for the local regions were created by Relion using maps containing the corresponding regions from the consensus map, which were extracted by the segmentation function of Chimera (*59*).

### Model building

The structural coordinates were built based on the cryo-EM structures of *S. scrofa* PEC [Protein Data Bank (PDB): 6GML] for RNAPII and DSIF (*11*), *Homo sapiens* nucleosome (PDB: 6A5O) for the histone proteins (*27*), *K. pastoris* RNAPII EC stalled at SHL(–5) (PDB: 6A5P) for the partially unwrapped nucleosome DNA (*27*), *K. pastoris* RNAPII EC stalled at SHL(–6) (PDB: 6A5O) for the intact nucleosome DNA (*27*), *S. scrofa* RNAPII complexed with *H. sapiens* TFIIS (PDB: 8A40) for TFIIS (*33*), and an arrested *Saccharomyces cerevisiae* RNAPII (PDB: 3PO2) for the backtracked part of RNA (*26*). The NELF coordinates were generated using AlphaFold2 (*60*). The generated model is consistent with the crystal structures of the NELF-BCE (PDB 8JJ6) (*61*) and NELF-AC (PDB 5L3X) (*62*) complexes. The RNAPII, TFIIS, NELF, and nucleosome parts were refined with phenix.real_space_refine (*63*), followed by manual editing with Coot (*64*), against focused maps containing the RNAPII, TFIIS, NELF, and nucleosome. The final models were generated by fitting the coordinates of each component into the whole-molecule maps, with manual adjustments of the connecting regions. All structural figures were prepared using UCSF Chimera (*59*) and PyMOL (Schrödinger; www.pymol.org).

The Materials and Methods should provide sufficient information to allow replication of the results. Begin with a section titled Experimental Design describing the objectives and design of the study as well as prespecified components.
