# Supplementary material for: Structural insights into promoter-proximal pausing of RNA polymerase II at +1 nucleosome
Source: Sci Adv. 2025 Mar 5;11(10):eadu0577. doi: 10.1126/sciadv.adu0577 (PMC11881899; doi:10.1126/sciadv.adu0577)
Supplement: Supplementary file 1 — Figs. S1 to S17 Tables S1 and S2 Legends for movies S1 to S4 [file sciadv.adu0577_sm.pdf]

Science Advances
AAAS

Supplementary Materials for

Structural insights into promoter-proximal pausing of RNA polymerase II at +1 nucleosome

Masahiro Naganuma *et al.*

Corresponding author: Shun-ichi Sekine, shunichi.sekine@riken.jp; Hitoshi Kurumizaka, kurumizaka@iqb.u-tokyo.ac.jp

Sci. Adv. **11**, eadu0577 (2025)
DOI: 10.1126/sciadv.adu0577

The PDF file includes:

Figs. S1 to S17
Tables S1 and S2
Legends for movies S1 to S4

Other Supplementary Material for this manuscript includes the following:

Movies S1 to S4

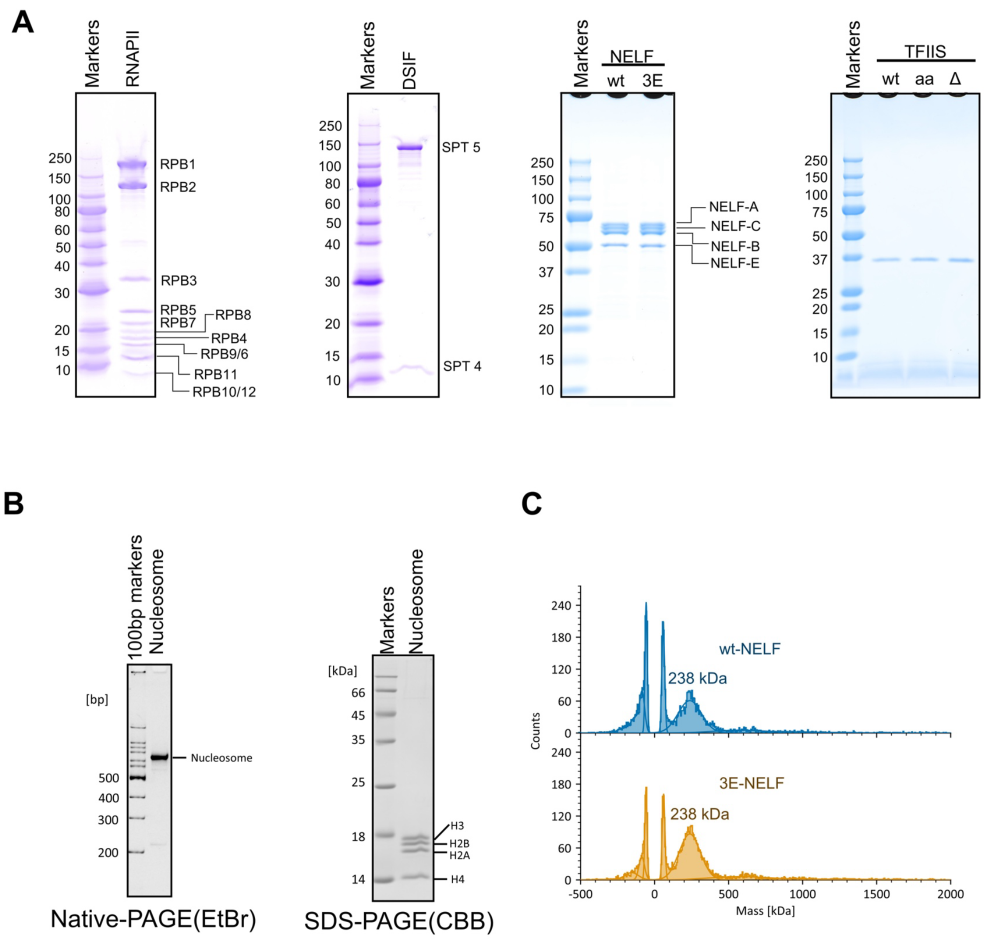

Fig. S1. Preparation of proteins used in this study. (**A**) The purified RNAPII, DSIF, NELF (wild-type and 3E-mutant), and TFIIS (wild-type and aa- and Δ-mutants) proteins were analyzed by 10-20% SDS-PAGE with Coomassie Brilliant Blue (CBB) staining. 3E denotes the NELF mutant, in which three Lys residues (K23, K27, and K34) of NELF-E are replaced with Glu. aa and Δ denote TFIIS mutants, in which two acidic residues essential for RNA-cleavage stimulating activity (D282 and E283) are replaced with Ala and deleted, respectively. (**B**) Native PAGE and SDS-PAGE gels for the reconstituted nucleosomes. (**C**) Mass photometry histograms of wild-type NELF (upper) and mutant 3E-NELF (lower). The histograms are fitted with Gaussian curves. The peaks at 238 kDa correspond to the molecular weight of the NELF heterotetramer complex.

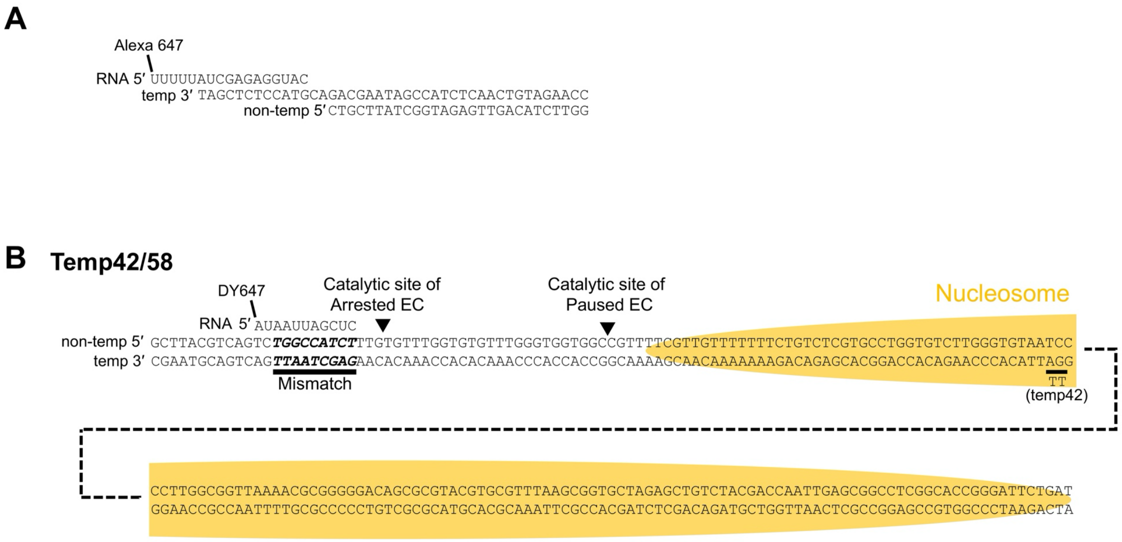

Fig. S2. The DNA/RNA scaffold and nucleosomal template. (**A**) Sequences of the DNA/RNA scaffold used in the electrophoretic mobility shift assay (EMSA, fig. S3B) and the transcription assays (fig. S3C,D). (**B**) Sequences of the temp42/58 nucleosomal template (*28*). The black arrowheads indicate the positions of the RNAPII catalytic site in the backtracked/arrested and paused EC structures. In the temp42 template, the bases at the indicated position were changed.

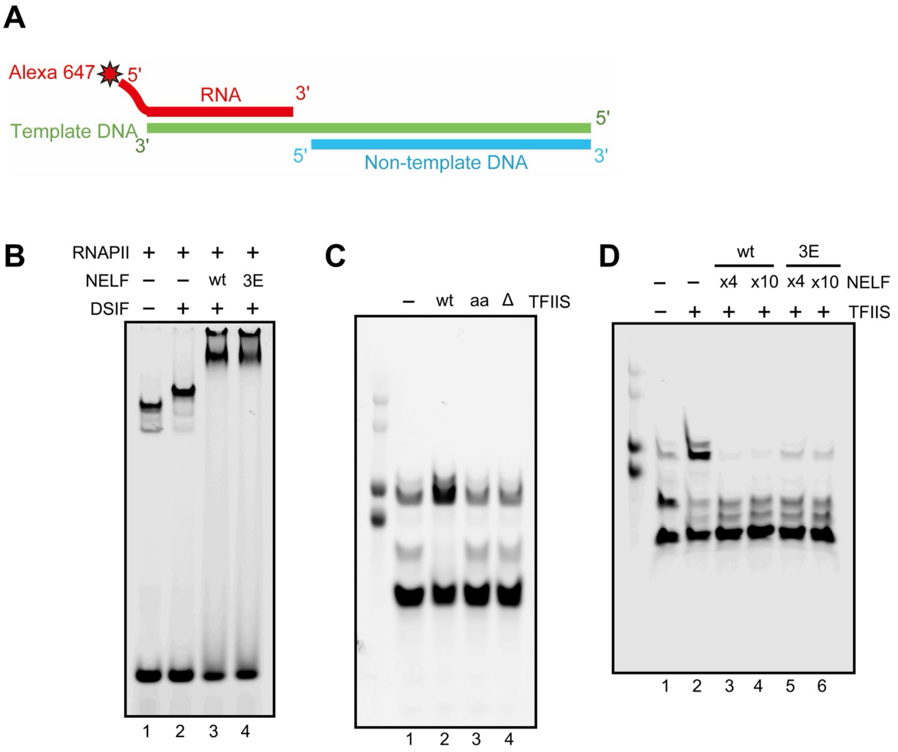

Fig. S3. The EC complex formation and transcription assays. (**A**) Schematic representation of the DNA/RNA scaffold. The template DNA, non-template DNA, and fluorescently-labeled primer RNA are colored green, cyan, and red, respectively. (**B**) Electrophoretic mobility shift assay (EMSA) of complexes formed on the DNA/RNA scaffold. 0.1 μM RNAPII, 0.4 μM DSIF, and 0.4 μM NELF were analyzed in the indicated lanes. (**C**) Urea PAGE analysis of RNA transcripts generated by transcription reactions in the absence or the presence of 0.1 μM TFIIS (wild-type, aa mutant, or Δ mutant). The reaction contained 0.1 μM RNAPII and 0.1 μM TFIIS (wild-type, aa mutant, or Δ mutant), as indicated. (**D**) Urea PAGE analysis of RNA transcripts generated by transcription reactions in the absence or presence of NELF (wild-type or 3E mutant). The reaction contained 0.1 μM RNAPII, 0.4 μM DSIF, 0.1 μM TFIIS, and 0.4 μM (x4) or 1 μM (x10) of NELF (wild-type or 3E mutant), as indicated.

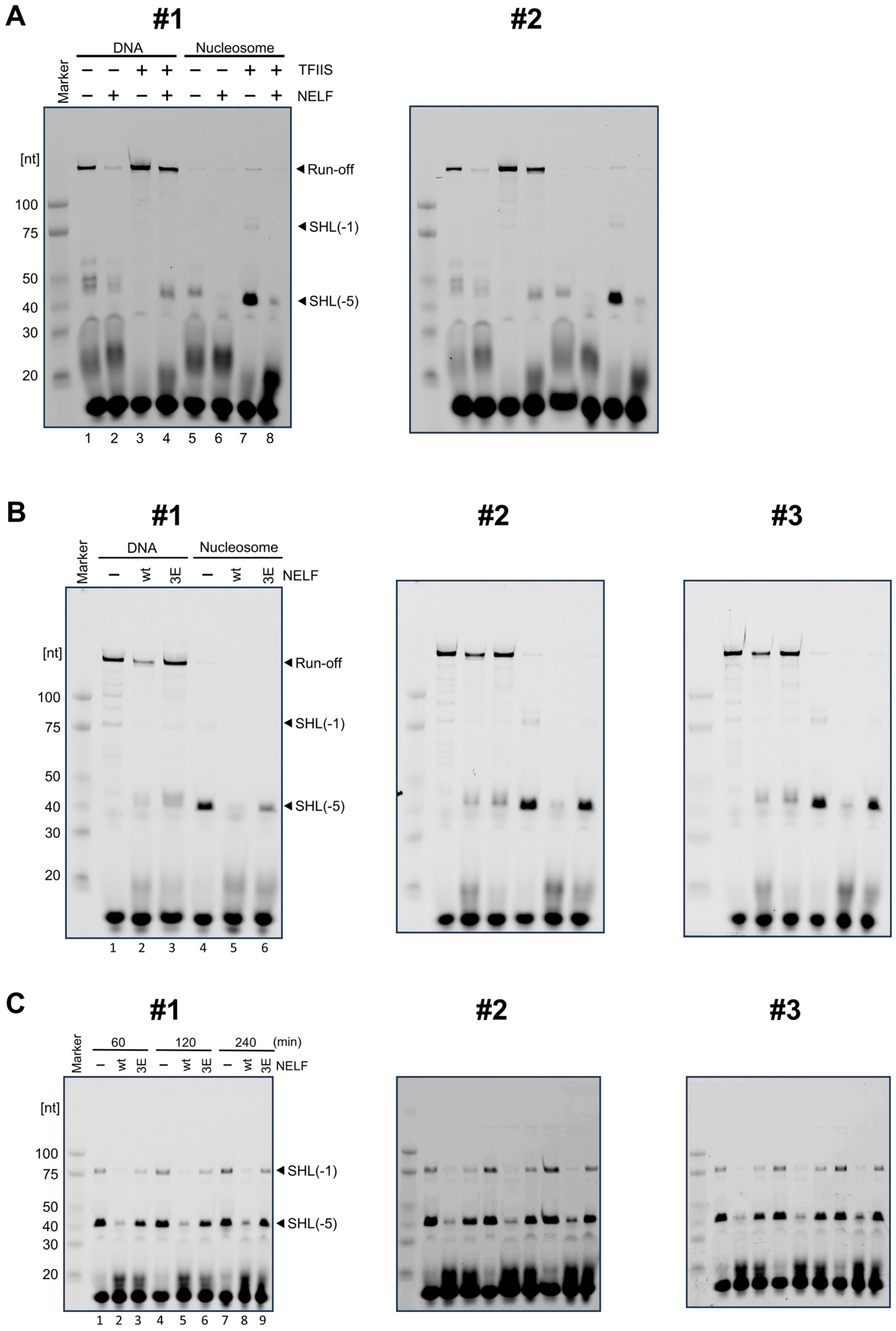

Fig. S4. Transcription assays. (**A**, **B**, **C**) Replications of transcription assays in Figs. 1B, 3C, and 4G are shown. The #1 images are the same as those in Figs. 1B, 3C, and 4G.

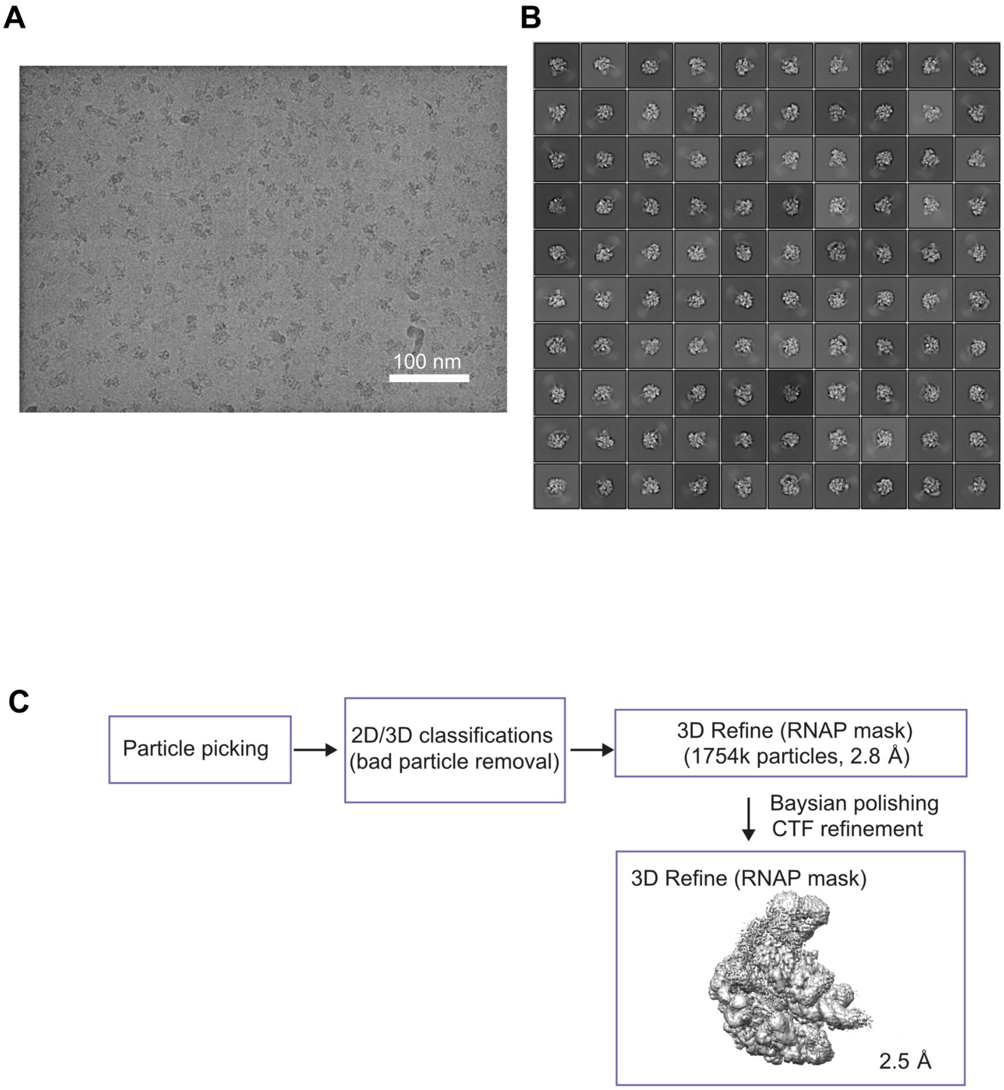

Fig. S5. Cryo-EM data collection and initial image processing of the dataset of EC without TFIIS. (**A**) Representative micrograph of the cryo-EM dataset. (**B**) Representative 2D class averages from the reference-free 2D classification calculated after removing bad particles. (**C**) Workflow of the initial stage of the image processing. The density map was generated by Chimera (*59*).

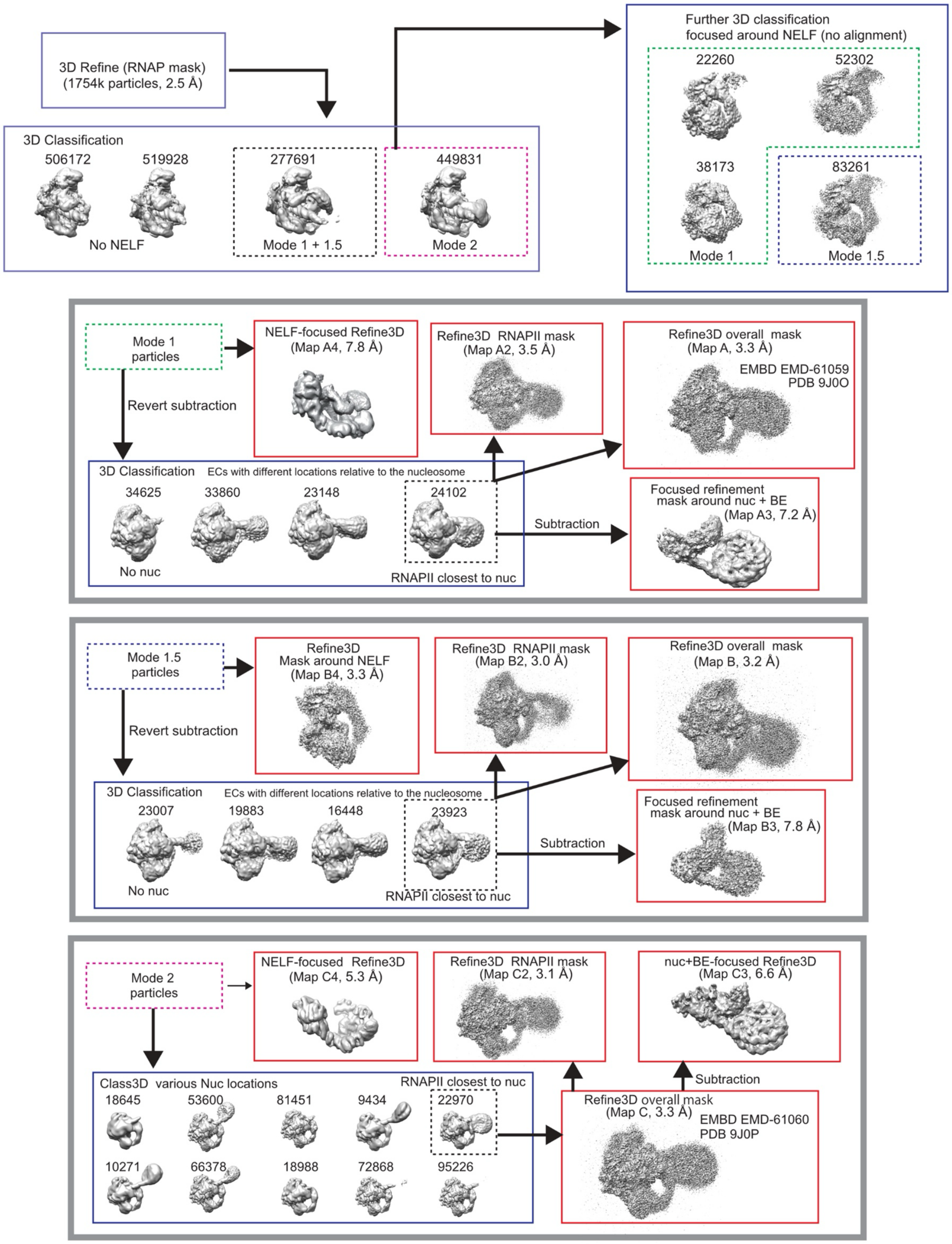

Fig. S6. Further classification of EC without TFIIS. The density maps were prepared by Chimera (*59*).

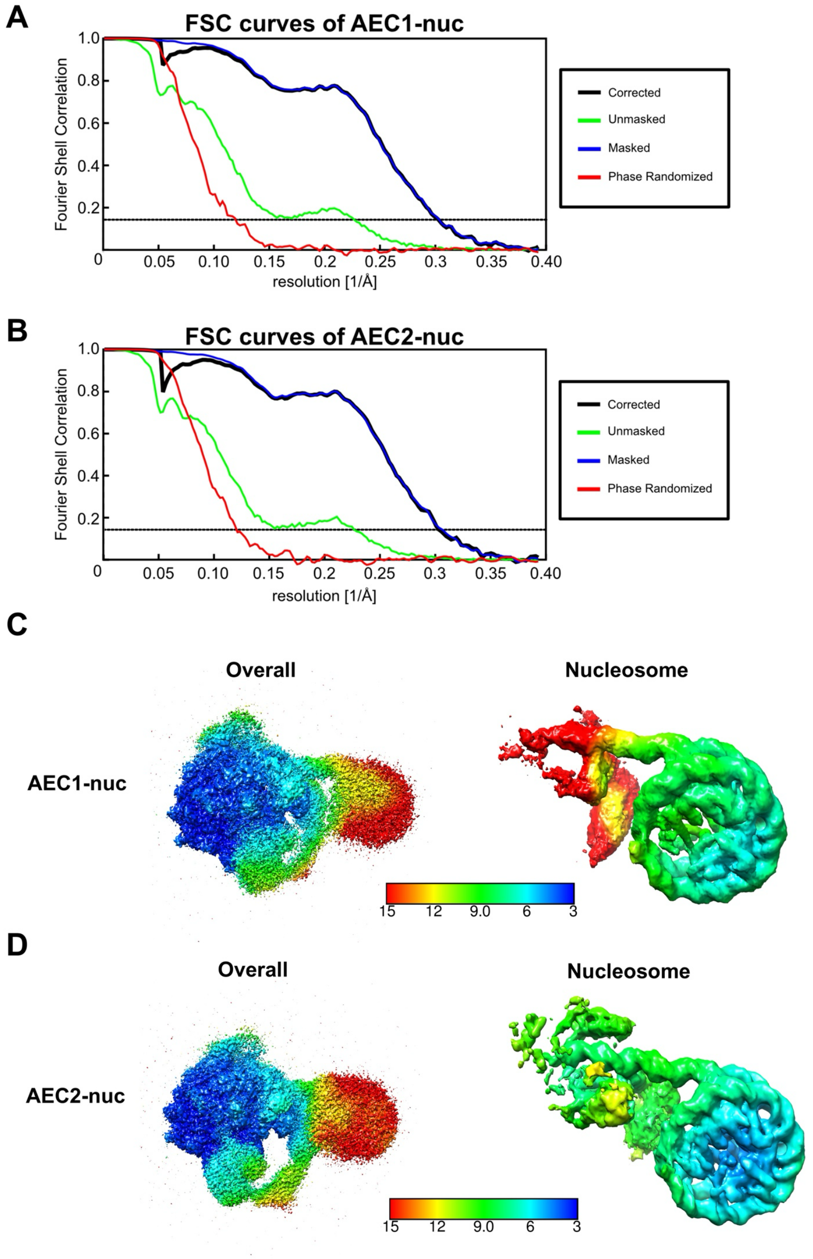

Fig. S7. Fourier shell correlation curves and local resolution maps for the RNAPII-nucleosome complexes and the nucleosomes. (**A**, **B**) Gold-standard Fourier shell correlation (FSC) curves of AEC1-nuc (**A**) and AEC2-nuc (**B**), calculated by Relion Refine3D (*55*). Dashed lines represent the FSC threshold of 0.143. (**C**, **D**) Cryo-EM maps of overall reconstructions (left) and nucleosome reconstructions after RNAPII subtraction (right) in AEC1-nuc (**C**) and AEC2-nuc (**D**) are shown. The maps are colored according to local resolution values. The local resolutions were calculated with RELION3.1 (*55*) and the maps were generated with Chimera (*59*).

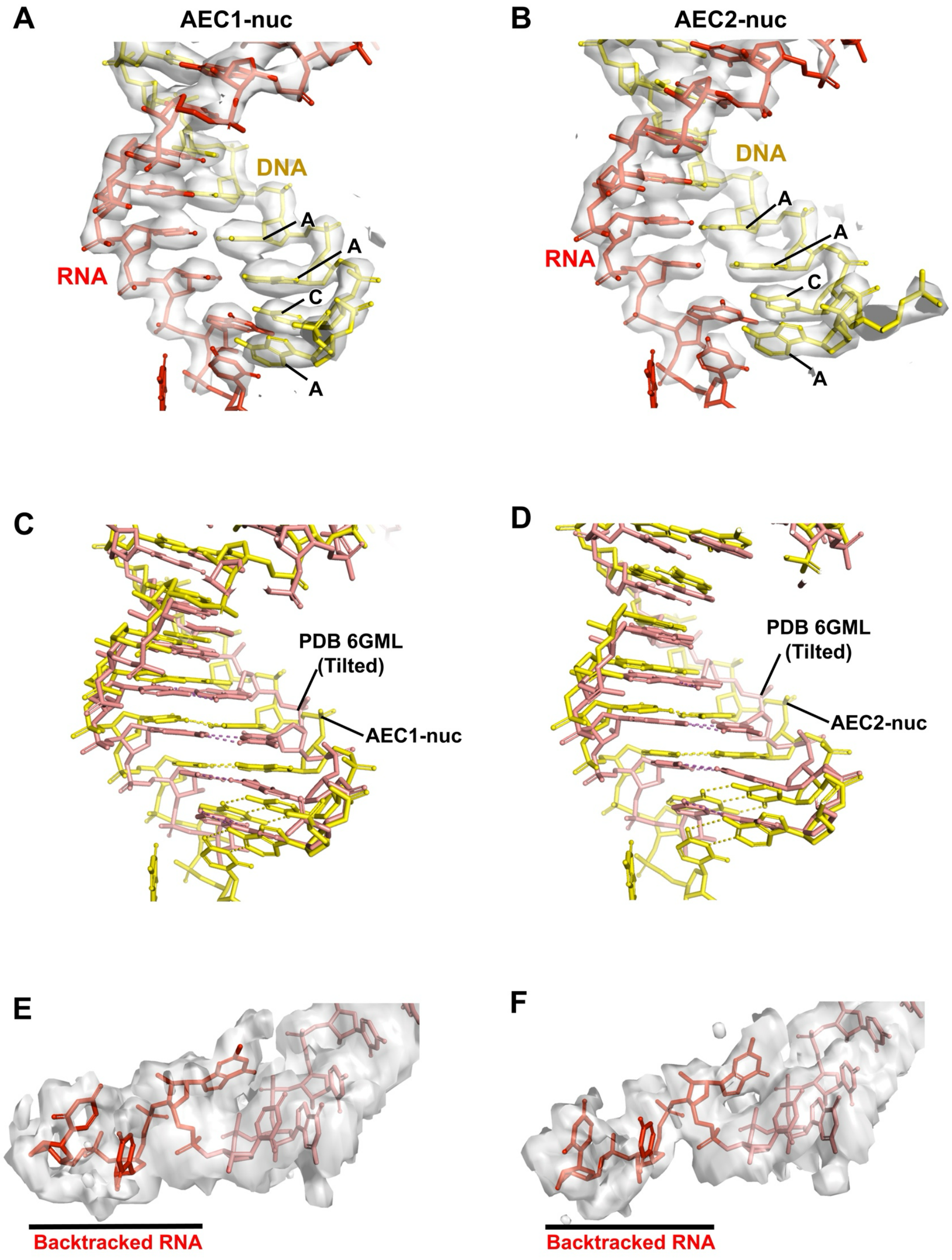

Fig. S8. The DNA/RNA hybrids in the active site of RNAPII. (**A**, **B**) The DNA/RNA hybrids in the RNAPII active site in AEC1-nuc (**A**) and AEC2-nuc (**B**) are depicted as yellow/red stick models, with the cryo-EM densities superimposed as transparent gray surface models. (**C, D**) The DNA/RNA hybrids in AEC1-nuc (**C**) and AEC2-nuc (**D**) are superimposed with the tilted-DNA/RNA hybrid in the previously reported PEC (PDB: 6GML). (**E**, **F**) Density maps of backtracked RNA in AEC1-nuc (**E**) and AEC2-nuc (**F**). The RNA molecules are shown as stick models. The density maps of the RNA molecules were extracted using UCSF Chimera and are shown as transparent gray surface models. The structural models were prepared using PyMOL.

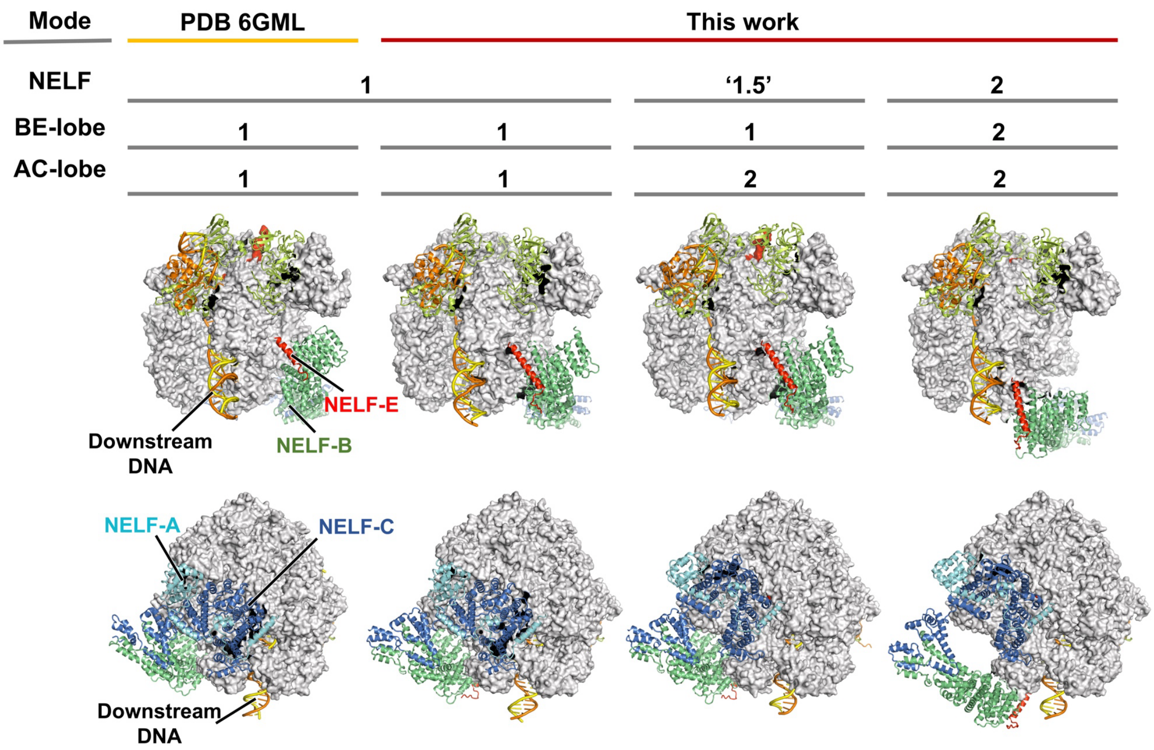

Fig. S9. Comparison of the NELF binding modes. Structures of paused EC (PDB: 6GML) and arrested/paused ECs (modes 1, 1.5, and 2, this study) are displayed in two orientations. The structural models were prepared using PyMOL.

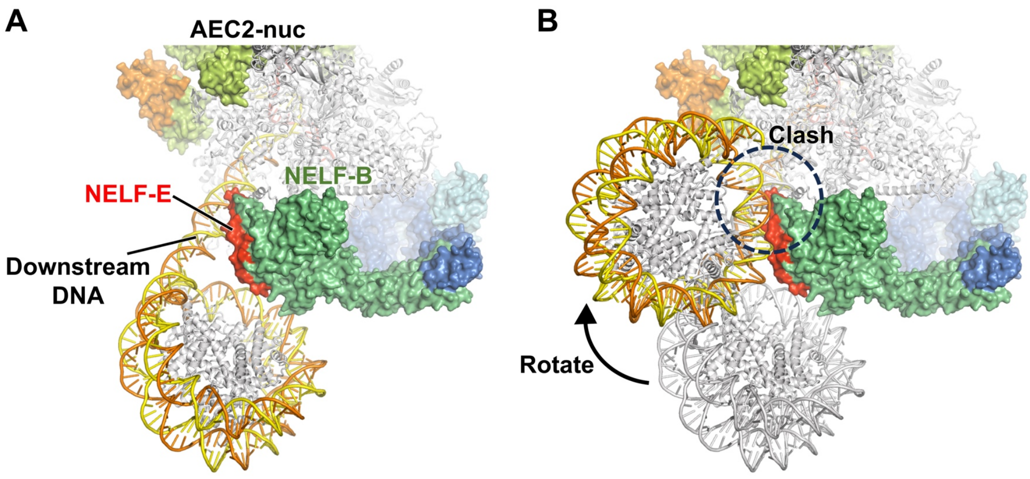

Fig. S10. Model of nucleosome rotation in front of EC. (**A**, **B**) Close-up view around the downstream nucleosome in AEC2-nuc. (B) If RNAPII translocates downstream by 6 bp, then the nucleosome could rotate around the downstream DNA axis by ~216º, and the rotated nucleosome would collide with NELF-E. The structural models were prepared using PyMOL.

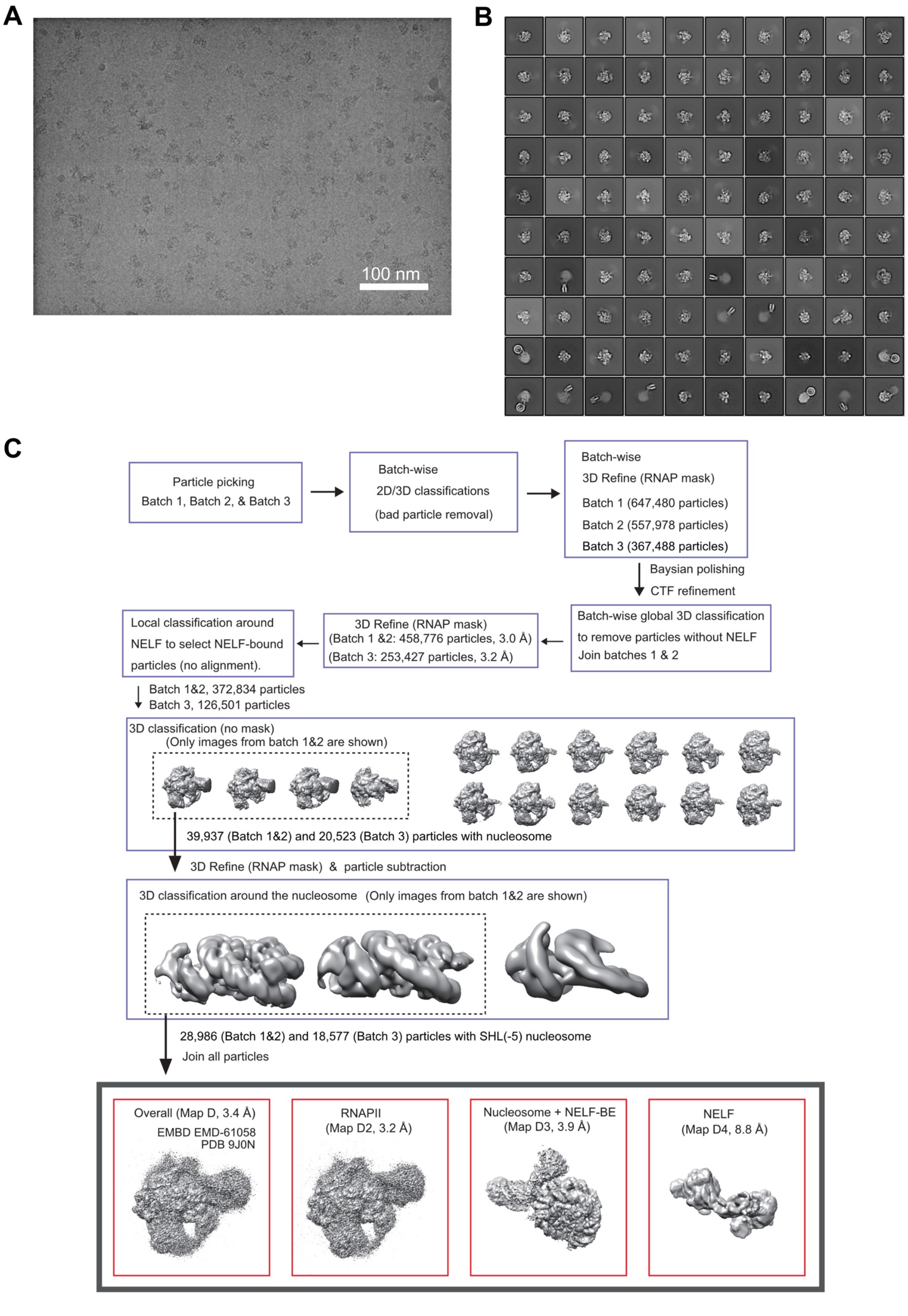

Fig. S11. Cryo-EM data collection and image processing of EC with TFIIS (PEC2-nuc). (**A**) Representative micrograph. (**B**) Representative 2D class averages from the reference-free 2D classification calculated after removing bad particles. (**C**) Workflow of the image processing. The density maps were prepared by Chimera (*59*).

A

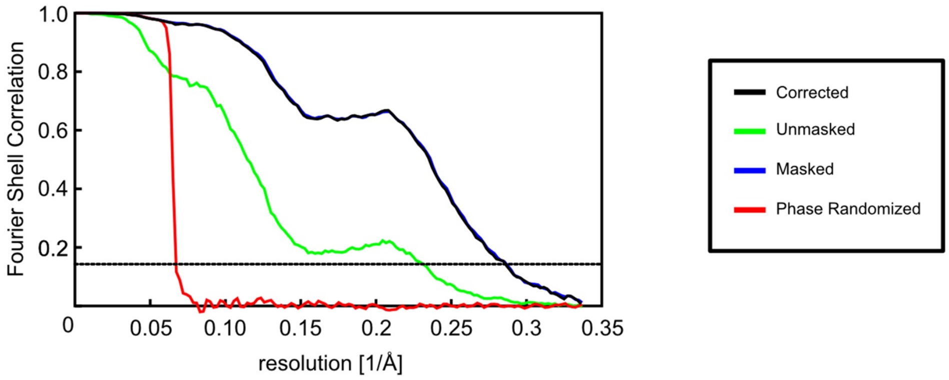

B

PEC2-nuc

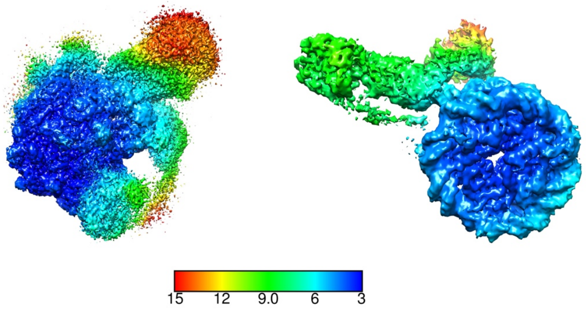

Fig. S12. Fourier shell correlation curves and local resolution maps for the RNAPII-nucleosome complexes and the nucleosomes. (**A**) Gold-standard Fourier shell correlation (FSC) curves of the overall reconstruction of PEC2-nuc. (**B**) Cryo-EM maps of overall reconstructions (left) and nucleosome reconstructions after RNAPII subtraction (right). The maps are colored according to local resolution values. The local resolutions were calculated by RELION3.1 (*55*) and the maps were generated by Chimera (*59*).

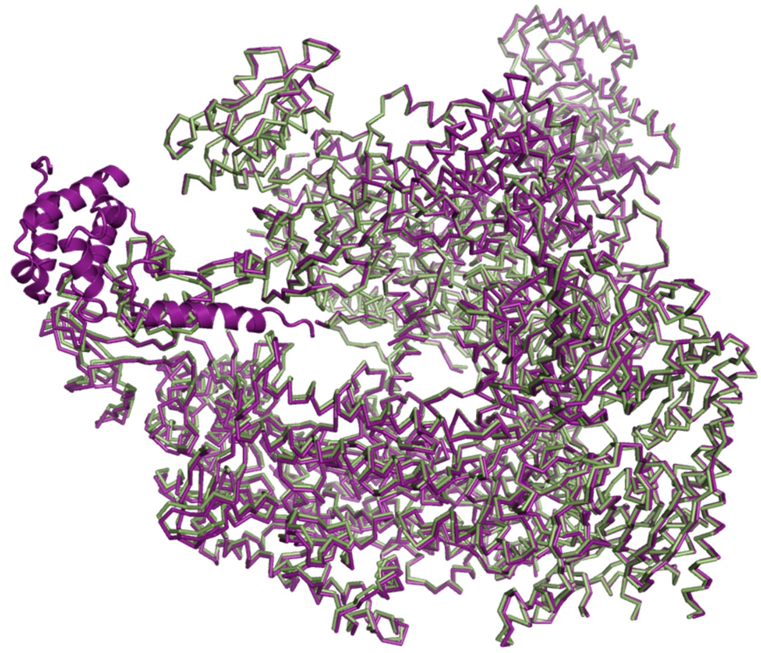

Fig. S13. Comparison of RNAPII structures with and without the bound TFIIS. The RNAPII structure in PEC2-nuc (with bound TFIIS) is superimposed with that in AEC2-nuc (without TFIIS). RNAPIIs in PEC2-nuc and AEC2-nuc are shown as purple and green trace models. TFIIS is shown as a purple cartoon model. The structural models were prepared using PyMOL.

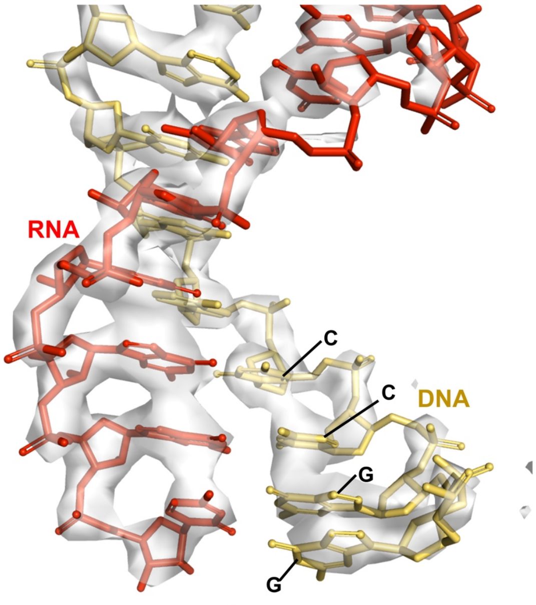

Fig. S14. RNAPII active site in PEC2-nuc. The DNA/RNA hybrid in the RNAPII active site superimposed with the cryo-EM density, shown as a transparent gray surface. The structural models were prepared using PyMOL.

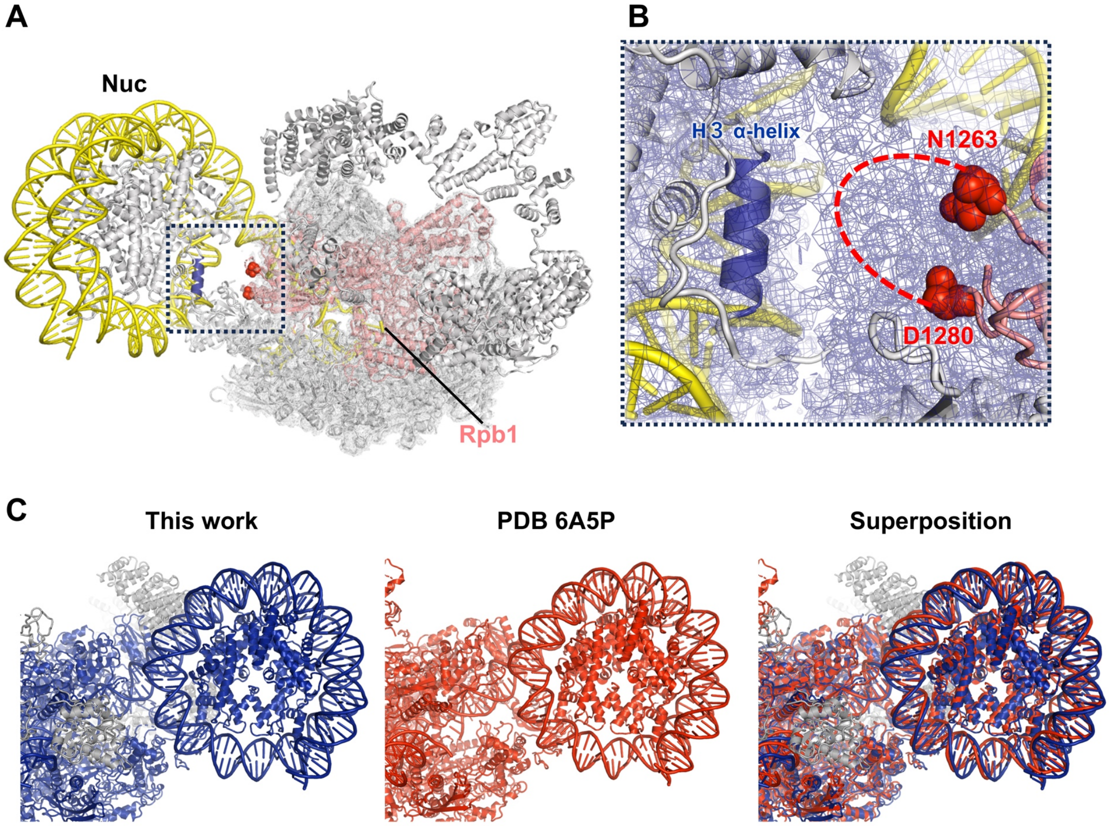

Fig. S15. The RNAPII-nucleosome interaction. (**A**) Overall structure of PEC2-nuc at the nucleosome. The RPB1 subunit of RNAPII is colored pink, while DNA is colored yellow. (**B**) Interactions between histone H3 and RPB1 at SHL(–5) of the nucleosome. Close-up view of the area enclosed by the square in panel A. N1263 and D1280 are depicted as red spherical models, and the disordered region is marked by a red dashed curve. The α-helix of H3 preceding the histone tail is represented by a blue ribbon model. The cryo-EM density is depicted as a blue mesh. (**C**) Close-up views of the RNAPII-nucleosome contact sites in the current PEC2-nuc structure (left), those in the RNAPII-nucleosome complex at SHL(–5) (PDB: 6A5P) (middle), and superimposition of the two (right). The structures, represented as cartoons or meshes, were prepared using PyMOL.

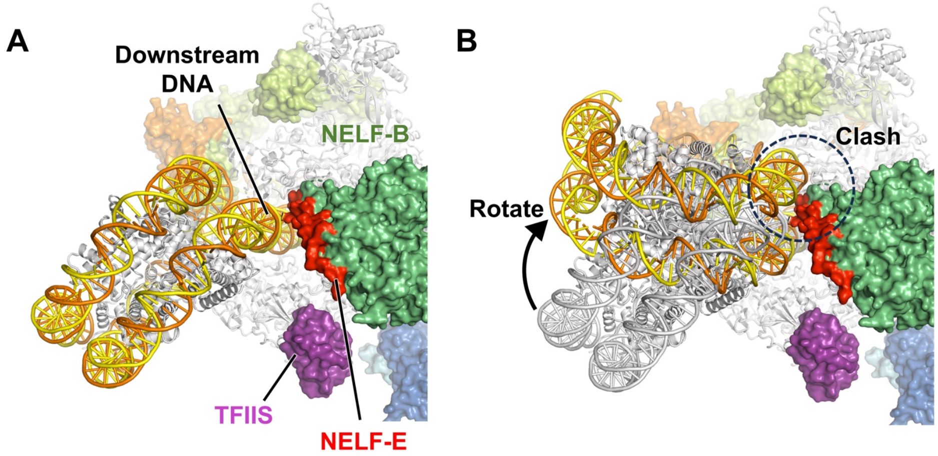

Fig. S16. Model of nucleosome rotation in front of PEC2-nuc. (**A**) Close-up view around the downstream nucleosome. NELF-E, NELF-B, and TFIIS are indicated. (**B**) If RNAPII translocates downstream by 2 bp, then the nucleosome could rotate around the downstream DNA axis by ~72°, and the rotated nucleosome would collide with NELF-E. The structural models were prepared using PyMOL.

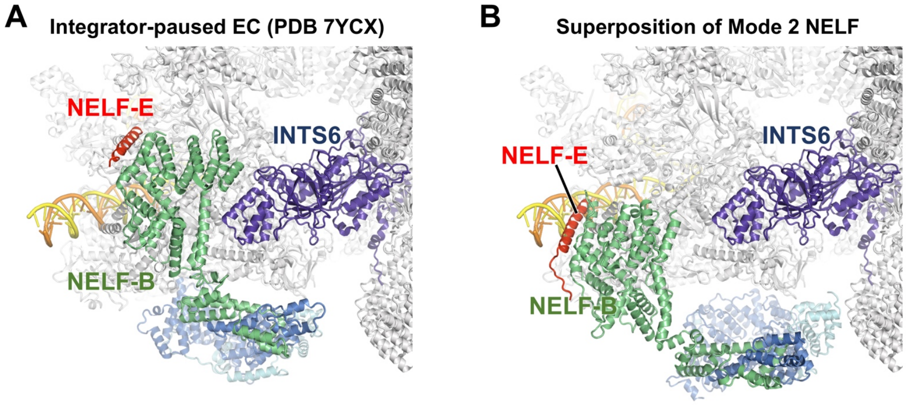

Fig. S17. The Integrator-EC interaction. (**A**) The interface between the Integrator INTS6 subunit and NELF-B in the paused EC (PDB 7YCX). (**B**) Superimposition of the NELF-BE lobe in mode 2. NELF-B, NELF-E, and INTS6 are represented by red, green, and purple ribbon models, respectively. The structural models were prepared using PyMOL.

Table S1. Data collection statistics.

Data collection	EC without TFIIS (batch1)	EC without TFIIS (batch2)	EC with TFIIS (batch1)	EC with TFIIS (batch2)	EC with TFIIS (batch3)
Microscope	Krios G4 (RIKEN BDR)	Krios G4 (RIKEN BDR)	Krios G4 (Univ.Tokyo, IQB)	Krios G4 (Univ.Tokyo, IQB)	Krios G4 (RIKEN BDR)
Voltage (kV)	300	300	300	300	300
Camera	K3/BioQuantum	K3/BioQuantum	K3/BioQuantum	K3/BioQuantum	K3/BioQuantum
Magnification	81,000	81,000	81,000	81,000	81,000
Pixel size (Å/pixel)	1.06	1.06	1.06	1.06	1.06
Total electron dose ($e^-/Å^2$)	53.9	60.4	60.5	62.8	58.4
Exposure rate (e^-/pixel/sec)	14.6	16.4	14.2	14.8	15.8
Exposure time (sec)	4.19	4.19	4.5	4.5	4.19
Number of frames	48	48	40	40	48
Defocus range (μm)	−1.0 to −2.5	−1.0 to −2.5	−1.0 to −2.5	−1.0 to −2.5	−1.0 to −2.5
Automation software	EPU	EPU	EPU	EPU	EPU
Energy filter slit width (eV)	15	15	20	20	15
Micrographs collected (no.)	8,789	34,874	17,602	15,501	22,408

Table S2. Refinement and model building statistics.

Refinement and Model building	AEC1-nuc (Mode 1)	AEC2-nuc (Mode 2)	PEC2-nuc (Mode 2)
Sample	EC without TFIIS	EC without TFIIS	EC with TFIIS
Nucleosome	Intact	Intact	Unwrapped to SHL(–5)
Image processing software	Relion	Relion	Relion
Final particles (no.)	24102	22970	47563
Pixel size for refinement (Å)	1.272	1.272	1.484
Symmetry imposed	C1	C1	C1
Resolution (global, Å)			
FSC 0.5 (unmasked/masked)	9.3/4.0	9.5/3.9	7.2/4.1
FSC 0.143 (unmasked/masked)	4.5/3.3	4.4/3.3	4.0/3.4
**Model composition**			
Protein residues	6504	6502	6623
RNA/DNA residues	387	387	335
Ligands	9 Zn, 1 Mg	9 Zn, 1 Mg	9 Zn, 1 Mg
**Model Refinement**			
Refinement package	Phenix (realspace)	Phenix (realspace)	Phenix (realspace)
R.m.s. deviations			
Bond lengths (Å)	0.004	0.003	0.006
Bond angles (º)	0.612	0.549	0.641
**Validation**			
MolProbity score	1.94	1.8	1.96
Clashscore	12.50	10.92	12.43
Rotamer outliers (%)	0.00	0.00	0.00
**Ramachandran plot**			
Favored (%)	95.29	96.29	94.81
Allowed (%)	4.69	3.69	5.19
Outliers (%)	0.02	0.02	0.0
PDB ID	9J0O	9J0P	9J0N
EMDB ID	EMD- 61059	EMD-61060	EMD- 61058

Movie S1. Structure of AEC1-nuc.

Movie S2. Structure of AEC2-nuc.

Movie S3. Structural transition between the NELF-binding modes 1 and 2.

Movie S4. Structure of PEC2-nuc.
